# Role of artificial intelligence in developing predictive models for major adverse cardiovascular outcomes using CCTA adipose tissue characteristics: a systematic review and meta-analysis

**DOI:** 10.1093/ehjdh/ztag104

**Published:** 2026-07-14

**Authors:** Sadaf Salehi, Seyed Hesam Hojjat, Ali Samadi Shams, Sepehr Ramezanipour, Armina Farkarian, Ali Azizi, Ghazaleh Kokabi Ghahremanpour, Hossein Movahed, Nikta Heidari, Soha Amiri, Aref Ghanaatpisheh

**Affiliations:** Student Research Committee, Iran University of Medical Sciences, Tehran, Iran; Faculty of Medicine, North Khorasan University of Medical Sciences, Bojnurd, Iran; Department of Cardiology, Islamic Azad University, Tabriz, Iran; Student Research Committee, Fasa University of Medical Sciences, Fasa, Iran; Student Research Committee, Shiraz University of Medical Sciences, Shiraz, Iran; Student Research Committee, Tabriz University of Medical Sciences, Tabriz, Iran; Hamidiye International School of Medicine, University of Health Sciences, Istanbul 3400, Türkiye; Student Research Committee, Jahrom University of Medical Sciences, Ostad Motahari Avenue, P.O. Box 193, Jahrom 74148-46199, Iran; Student Research Committee, Jahrom University of Medical Sciences, Ostad Motahari Avenue, P.O. Box 193, Jahrom 74148-46199, Iran; Student Research Committee, Jahrom University of Medical Sciences, Ostad Motahari Avenue, P.O. Box 193, Jahrom 74148-46199, Iran; Student Research Committee, Jahrom University of Medical Sciences, Ostad Motahari Avenue, P.O. Box 193, Jahrom 74148-46199, Iran; Cardiology Department, Jahrom University of Medical Sciences, Ostad Motahari Avenue, P.O. Box 193, Jahrom 74148-46199, Iran

**Keywords:** AI, Predictive model, Adipose tissue, CCTA, Cardiovascular disease

## Abstract

Cardiovascular diseases remain as a leading cause of mortality and morbidity worldwide, with coronary artery disease (CAD) and its complications, collectively referred to as major adverse cardiovascular events (MACEs), necessitating accurate risk stratification. Coronary computed tomography angiography (CCTA) has emerged as a valuable non-invasive imaging modality, and adipose tissue characteristics derived from CCTA have shown promise as imaging biomarkers for MACE prediction. This systematic review and meta-analysis aimed to evaluate the predictive performance of artificial intelligence (AI)-driven models incorporating CCTA-derived adipose tissue radiomic features for forecasting MACEs. A systematic review and random-effects meta-analysis were conducted in accordance with the Preferred Reporting Items for Systematic Reviews and Meta-Analyses guidelines. Studies evaluating predictive models developed using different AI algorithms that utilized adipose tissue radiomics as the primary predictor of MACEs in patients undergoing CCTA were included. Model performance was assessed using pooled area under the receiver operating characteristic curve (AUC), sensitivity, and specificity. Eleven studies comprising 47 244 participants were included in the analysis. AI-based models integrating adipose tissue radiomic features with clinical data consistently outperformed conventional risk assessment tools, with pooled AUCs ranging from 82.2% to 87.9%. Among the evaluated approaches, deep learning models demonstrated superior predictive performance compared with traditional machine learning and logistic regression-based models. However, considerable heterogeneity (*I*² > 96%) was observed across studies, reflecting variations in study design, imaging protocols, and AI methodologies. While AI-enhanced CCTA-based adipose tissue characterization demonstrates considerable potential for improving MACE risk prediction, methodological heterogeneity and limited external validation currently restrict its clinical applicability. Future research should prioritize standardized imaging and analytical methodologies, rigorous clinical and external validation, and transparent reporting to facilitate reliable integration of these AI models into routine cardiovascular risk assessment.

## Graphical Abstract

**Graphical Abstract:**
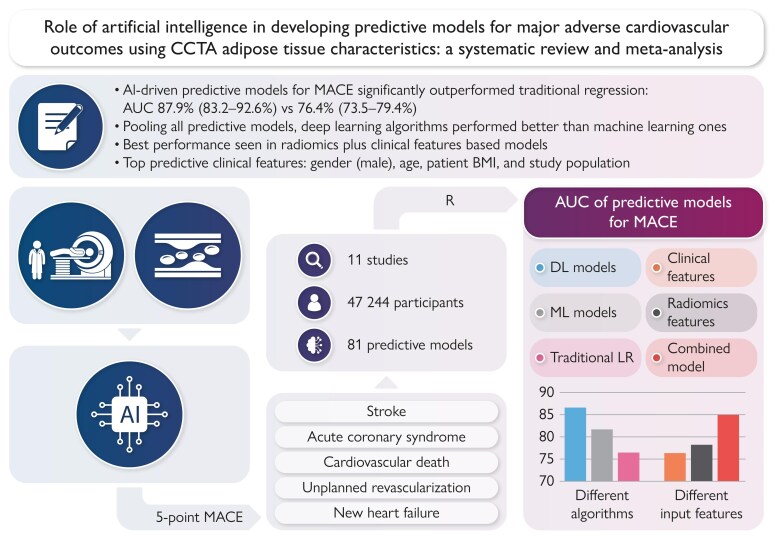

## Introduction

Cardiovascular diseases (CVDs) remain the primary cause of death and disability worldwide. From 1990 to 2019, annual mortality attributed to these diseases had a dramatic rise from 12.1 to 17.9 million.^1^ Besides this massive impact on global mortality, CVDs impose widespread disability and economic burdens.^2–4^ Among different cardiovascular conditions, coronary artery disease (CAD) is recognized as a significant cause of death and disability despite the current preventive strategies. CAD is characterized by the accumulation of atherosclerotic plaque within coronary arteries, which causes impaired blood flow, leading to angina or infarctions. This plaque accumulation may also happen in other major organ arteries, leading to various severe outcomes collectively known as major adverse cardiovascular outcomes (MACE), which refer to a broad spectrum of events, including myocardial infarction, stroke, heart failure, ischemic derived revascularization, and cardiac death.^5–7^ Studies show that only one-third of the MACE cases had previously experienced obstructive CAD symptoms.^8^ The profound global health burden posed by CVDs, as well as their clinical diagnostic challenges, especially in specific subgroups such as diabetics^8^ and women,^9^ creates a need for imaging tools both for timely diagnosis and risk stratification of population.

While invasive coronary angiography (ICA) is classically used to identify coronary stenosis, its cost and invasive nature limit its utility.^10,11^ Nowadays, as the role of imaging techniques has become increasingly vital, coronary computed tomography angiography (CCTA) has emerged as a cost-effective non-invasive modality in the diagnosis of possible CAD, which demonstrates strong concordance with ICA as the gold standard in assessing stenosis severity.^12,13^ CCTA offers valuable insights into identifying pathologic features indicating plaque vulnerability, elevating the risk of MACE.^14^ Besides plaque features, CCTA also shows adipose tissue characteristics, including average CT attenuation, a marker of inflammation, and tissue composition.^15,16^ Previous research highlights the role of adipose tissue depots, including perivascular adipose tissue (PVAT), the fat surrounding coronary arteries, and epicardial adipose tissue (EAT), the adipose layer located between the myocardium and visceral pericardium, in the pathogenesis of CVDs.^15,17^

Inflammation in coronary arteries has been observed to alter adipogenesis in perivascular fat, resulting in lower lipid content in adipocytes around the inflamed vessels and detectable changes in adipose tissue CT attenuation.^18^ Regarding these, novel radiologic biomarkers have emerged to identify this inflammatory process at more initial steps for a timely intervention.^19^ Fat attenuation index (FAI) is one of these markers derived from adipose tissue in CCTA, which has shown promise for detecting vascular inflammation that was not evaluated in the conventional methods. FAI is also associated with plaque instability and helps in early diagnosis of subclinical CAD.^18,20,21^ However, manual measurement of FAI is both time-consuming and error-prone, limiting its clinical application and emphasizing the need for enhanced qualitative and quantitative methods.^22^

Artificial intelligence (AI), which includes techniques such as machine learning (ML) and Deep learning (DL), offers substantial benefits in the field of cardiac imaging.^23^ First, AI algorithms enhances CCTA capabilities, improving imaging processes, segmentation, and extracting biomarkers.^8,24,25^ It also enables us to integrate radiomics, an advanced technique for acquiring quantitative imaging features, with our diagnostic and prognostic models.^26^ Furthermore, AI could be used for model training, and many studies have shown promising performance of AI-based models by handling large datasets and complex nonlinear associations.^27^ Recent studies demonstrated that models trained by AI algorithms could significantly enhance the detection and prediction of MACEs.^28,29^ However, key obstacles, including variability in imaging techniques, potential limitations in generalizability from single-centre studies, challenges with interpretability of these models, and insufficient validation in some of those, remain barriers to widespread adoption in everyday practice.

In this review, we evaluate AI-based models’ role in improving the early detection and risk stratification of MACEs using adipose tissue segments defined by CCTA. By integrating radiomics features derived from adipose tissue with some clinical and conventional radiologic-based features and comprehensively investigating these new models across different studies and various populations, this review aims to provide clinicians with robust evidence for identifying high-risk patients, bridging existing diagnostic gaps, and advancing toward innovative, personalized approaches for more accurate prediction and prevention of MACEs.

## Methods

This systematic review was conducted based on the Preferred Reporting Items for Systematic Reviews and Meta-Analyses (PRISMA) guidelines,^30^ and the study protocol was registered in the Open science framework (OSF) (https://doi.org/10.17605/OSF.IO/HK37P).

### Study selection

Studies evaluating performance metrics of AI models trained by adipose tissue features predicting MACE occurrence in patients undergoing CCTA were aimed to be assessed in this study. Using related keywords as illustrated in Supplementary material online, *Table S1*, a systematic search was performed across PubMed/MEDLINE, Scopus, Web of Science, and Embase up to 27 November 2024. As Google Scholar is more of a search engine rather than a research database, and tailoring a specific search string for this engine is more challenging, results from the initial search in this source at the same timeframe of above databases were evaluated by the person performing the search and only the first 158 results, which seemed more related to our research topic, were imported to reference manager software. After automatically deleting duplicate records, an initial screening based on the record's title and abstract was performed using Rayyan software,^31^ followed by a full-text screening. In each screening phase, records were simultaneously assessed by two independent researchers in a blinded manner, and yet another reviewer resolved the conflicts. Our eligibility criteria included all English peer-reviewed original studies involving human participants of any age, sex, or ethnicity, with or without pre-existing cardiovascular diseases. We aimed to focus on studies that applied AI methodologies in developing predictive models for MACE occurrence, ensuring relevance to our research objectives. Various definitions for MACE have been suggested in the literature. To comprehensively review the current state of AI in MACE prediction, we utilized a five-point MACE definition as discussed by Bosco *et al*., encompassing: acute myocardial infarction, stroke, cardiovascular death, unstable angina, unplanned revascularization events, and heart failure.^7^ Review articles, book chapters, editorials, studies with unrelated scope, conference presentations without a further peer-reviewed full text, and commentary articles were excluded as they did not provide primary data for our analysis. This selection criterion ensured that only studies directly contributing to original research were included.

### Data extraction and management:

The data included in the paper were extracted independently by four reviewers using a custom-made extraction Excel sheet. Extracted information includes study characteristics (first author's name, year of publication, country, study design, patients demographics, outcome), Imaging characteristics (details of scanner parameters, segmentation methods, radiologic and radiomics extracted features, adipose tissue location), and utilized AI algorithms as well as model details (e.g. AI class, feature selection method, performance metric, clinical validity, and interpretability measurements).

For model performance, we extracted only summary evaluation metrics reported for out-of-sample evaluation (external test set when available; otherwise independent validation or cross-validation). Training-set performance metrics, if reported, were recorded only descriptively to assess potential optimism and were not used in any pooled meta-analytic estimate.

### Assessment of risk of bias in included studies:

Two independent reviewers conducted the risk of bias assessment for included records in this study using PROBAST-AI,^32^ a modified version of the PROBAST questionnaire published in 2025 which was designed explicitly for prognostic studies utilizing AI algorithms in their predictive modelling. This tool evaluates the risk of bias in four domains: participants, predictors, outcome, and analysis. Furthermore, it helps researchers assess applicability of included studies regarding three first domains for the main research question.

### Statistical analysis

Statistical methods and effect size selection followed guidance from the *Cochrane Handbook for Systematic Reviews of Interventions*.^33^ Meta-analysis was performed using a random-effects framework. Pooled estimates with 95% confidence intervals (CIs) were computed for AUC, and when available, for accuracy, as well as sensitivity and specificity.

#### Unit of analysis and data extraction

The analytic unit was a *model evaluation*, defined as one distinct performance estimate reported for a specific algorithm–feature set–dataset split within a study. We defined the *dataset* as the cohort reported in each included study (*Table 1*), including any internal split (training/validation/test) and any external validation cohort when available. Each distinct AUC (with its 95% CI) reported for a given model and split was extracted as a separate effect size. When studies reported AUCs separately for development (training/cross-validation) and validation/test cohorts, these were extracted and analyzed as separate model evaluations; this yielded *k* = 74 AUC effect sizes for the ‘all model evaluations’ meta-analysis. As out-of-sample performance is most clinically relevant, test/external validation results were favoured when present; however, all eligible evaluations (including development/training or cross-validation when separately reported) were included in the ‘all model evaluations’ synthesis.

**Table 1:** Summary of main characteristics of included studies

Author	Location	Study type	Study design	Sample size	follow-up (month)	% Male	Age (mea*n* ± SD)	Final outcome	Outcome incidence	time to MACE (month)
**Zhang *et al*. (2024)**	China	Single-centre	Case-control	282	50.4	61.93	60.23 ± 9.17	Total MACE	113 / 282	15.88
								MI	26/282	
								PCI	103/282	
								CABG	9/282	
								Cardiac death	3/282	
**Miao *et al*.** ^34^	China	Single-centre	Retrospective cohort	479	NR	65.76	66.85 ± 11.42	CAD	258/479	NR
**He *et al*.** ^35^	China	Single-centre	Prospective cohort	1077	36	64	62.58 ± 11.63	Total MACE	228 / 1077	NR
**Chan *et al*.** ^8^	united kingdom	Multi-centre	Prospective cohort	43884	13.44	53.1	58.69	Total MACE	5013/43 884	NR
								Non-fatal MI	2195/43 884	
								New HF	2040/43 884	
								Stroke	778/43 884	
								Cardiac death	2093/43 884	
**Militello *et al*.** ^36^	Italy	Single-centre	Retrospective cohort	118	NR	71.18	60.33 ± 13.2	CAD	118	NR
**You *et al*.** ^37^	China	Single-centre	Retrospective study	288	NR	52.34	59.81	Total MACE	144/288	11.82
**Wei He *et al*.** ^38^	China	Single-centre	Retrospective cohort	261	NR	72.03	63 ± 8	CAD(PCI)	105/261	NR
**Oikonomou *et al*.** ^29^	UK, USA, Germany	Multi-centre	Case–control	202	57.6	66.3	63.64 ± 12.78	Total MACE	101/ 202	NR
								Cardiac mortality	61/202	
**Huang *et al*.** ^28^	China	Single-centre	Retrospective cohort	100	36	NR	NA	CAD (ACS)	50 / 100	NR
**Zhan *et al*.** ^39^	China	Single-centre	Retrospective cohort	239	12	54.4	63.95 ± 10.78	Total MACE	47/239	NR
								Stroke	9/239	
								CHD (Conjestive)	10/239	
								Malignant arrhythmia	8/239	
								MI	20/239	
**Huang *et al*.** ^40^	China	Multi-centre	Retrospective cohort	314	12	74	60.84	Total MACE	60/274	NR

MACE, major adverse cardiovascular event; MI, myocardial infarction; PCI, percutaneous coronary intervention; CABG, coronary artery bypass graft; HF, heart failure; CAD, coronary artery disease; NR, not reported; CHD, congestive heart disease.

#### Primary and secondary meta-analytic datasets

We conducted meta-analyses on: (i) all eligible model evaluations across studies and splits (‘all model evaluations’), and (ii) the best-performing model evaluation per study, preferentially selected from the test/external validation split when available (otherwise, the most out-of-sample evaluation reported).

#### Pooling of AUC and accuracy

AUC was the primary discrimination effect size. For each AUC, standard errors (SEs) were derived from reported 95% CIs using

$SE=\frac{\left(\mathrm{Upper}\;95\text{\%}\;\mathrm{CI}-\mathrm{Lower}\;95\text{\%}\;\mathrm{CI}\right)}{2\times 1.96}.$ Pooled AUCs were calculated using inverse-variance weighting under a random-effects model, with weights


$$w=\frac{1}{\left(SE^{2}+\tau^{2}\right)},$$

where $\tau^{2}$ denotes the between-effect (between-study/model-evaluation) variance. Between-effect variance was estimated using the DerSimonian–Laird method. When pooled, accuracy was synthesized using the same univariate inverse-variance random-effects approach.

#### Pooling of sensitivity and specificity

For classification performance, sensitivity and specificity were jointly synthesized using a bivariate random-effects model (bivariate linear mixed model) to account for their correlation and to produce pooled sensitivity and specificity with 95% CIs.

#### Assessment of heterogeneity and prediction interval

Statistical heterogeneity was assessed using Cochran's *Q* (with heterogeneity considered statistically significant at *P* < 0.05), *I*^2^, and $\tau^{2}$. *I*^2^ was interpreted as negligible (0%–40%), moderate (30%–60%), substantial (50%–90%), and considerable (75%–100%) heterogeneity. For pooled AUC estimates, a 95% prediction interval (PI) was also calculated to indicate the range of effects expected in a future comparable study.

#### Publication bias

Potential publication bias/small-study effects were evaluated using Egger's regression test, with statistical significance defined as *P* < 0.05.

#### Subgroup analyses and meta-regression

To compare outcomes across different model types and explore potential sources of heterogeneity, we conducted subgroup meta-analyses and meta-regression. To address heterogeneity related to predictor inputs, models were categorized *a priori* into three modality groups: image-only (CCTA imaging/radiomics features without clinical predictors), tabular-only (clinical predictors/risk scores without imaging radiomics inputs), and multimodal image + tabular (combined imaging and clinical predictors). Subgroup analyses were performed accordingly.

#### Robustness and influence (leave-one-out analysis)

To evaluate robustness and identify influential data points, we conducted a leave-one-out (LOO) meta-analytic influence analysis (not subject-level cross-validation). Because individual participant data were not available, LOO was performed at the level of the effect size (model evaluation). For the ‘all model evaluations’ dataset, we sequentially omitted one extracted AUC effect size at a time and recalculated the pooled AUC under a random-effects model (DerSimonian–Laird). For the ‘best-performing model per study’ dataset (one selected model evaluation per study), this procedure is equivalent to leave-one-study-out. For each iteration, updated pooled AUC (95% CI), $\tau^{2}$, and *I*^2^ were recorded; the range of these estimates was used to assess stability of the summary effect.

All statistical analyses were conducted using Comprehensive Meta-Analysis (CMA) v4, R (v4.4.2), and Python (v3.11).

### Results

#### Overview of included studies

A total of 1584 records were identified through different databases, as illustrated in Supplementary material online, *Figure S3* of Supplementary Files. After removing 710 duplicate records, 874 unique records were screened by title and abstract. Of these, 702 studies were excluded for not meeting the inclusion criteria in the initial screening phase, and 172 full-text reports were sought for retrieval. Following full-text assessment, 148 reports were excluded for the following reasons: 4 due to languages other than English, 79 due to irrelevance to the study scope, and 64 due to wrong publication type. Since the focus of our review is on predictive models built using ML or DL algorithms, 13 other studies that used AI algorithms only for feature extraction and imaging quality enhancement were also excluded, and ultimately, 11 studies were deemed eligible and included in this systematic review and meta-analysis (see Supplementary material online, *Figure S3*  Supplementary Files).

**Figure 1.**
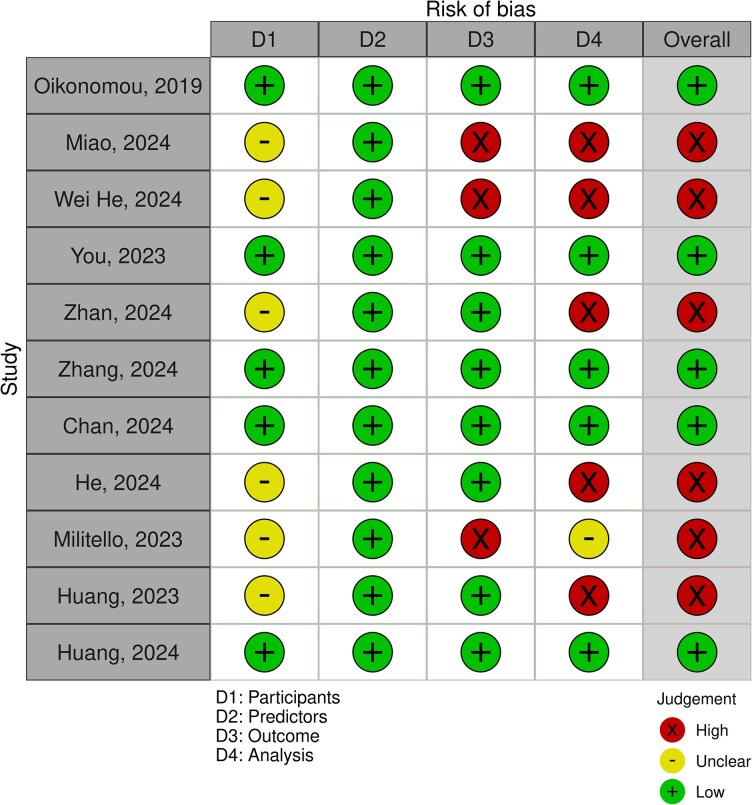
Risk of bias assessment in selected studies.

**Figure 2.**
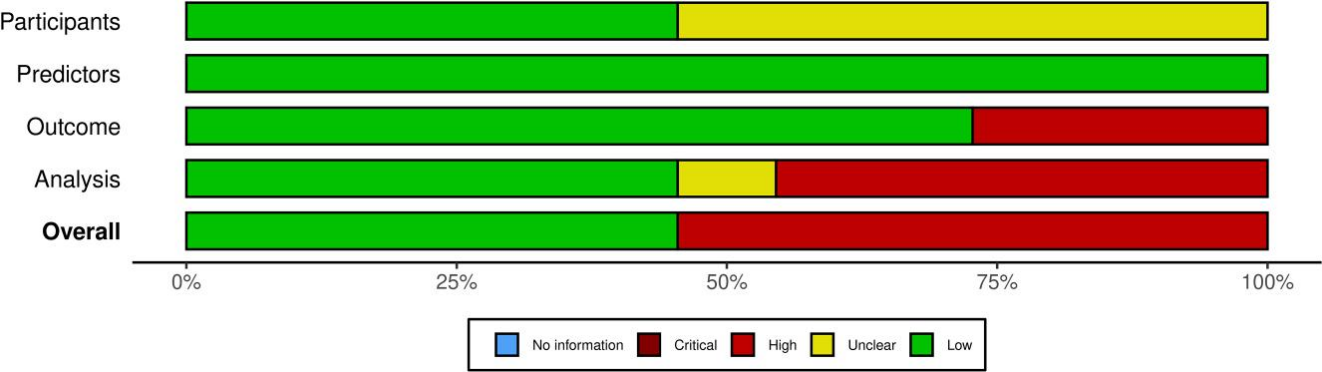
Stacked bar chart showing the proportion of included studies rated as having low (green), unclear (yellow), high (red), critical (dark red), or no information (blue) riskof bias across the four PROBAST domains: participants, predictors, outcome, and analysis, as well as the overall risk of bias assessment. Percentages represent the proportion of studies assigned to each risk category within each domain.

##### Study characteristics and demographics

Our review identified 11 studies encompassing 47244 participants, mainly across Asia and Europe. Seven were retrospective cohort studies,^28,34,36–40^ two were case–control studies,^8,29^ and two were prospective cohorts.^8,35^ The included studies evaluated the performance of various models for predicting CAD and MACE using a spectrum of features selected from clinical and radiological characteristics. The studies varied in design, with *Eight* (72.7%) being single-centre^25,28,34–39^ and *Three* (27.3%) multi-centre.^8,29,40^ Their follow-up period ranged from less than 12 to more than 57 months. The reported weighted mean of participants’ age and BMI were 58.8 years and 25.21, respectively. Common cardiovascular risk factors noted in the studies were defined as hypertension (HTN), myocardial infarction (MI), hyperlipidemia, diabetes mellitus (DM)/hyperglycemia, smoking, history of Cardiac interventions, personal and family history of CAD, and alcohol consumption. Common medications used among the studies’ participants were anti-platelets, statins, beta-blockers, angiotensin-converting enzyme inhibitors (ACEIs), angiotensin receptor blockers (ARBs), and calcium channel blockers (CCBs).

##### CCTA and adipose tissue measurements:

All studies utilized CCTA as their primary modality. Adipose tissue segmentation was performed manually or using auto-segmentation softwares like CoronaryDoc,^25,39^ Pyradiomics,^34,36,40^ EATseg,^37^ Autoplaque,^38^ SlicerRadiomics,^29^ and Deepwize.^35^ Most studies used perivascular adipose segments either around all three major cardiac vessels or RCA. One study compared the accuracy of models constructed based on epicardial vs. perivascular adipose tissue.^37^ Conventional adipose tissue characteristics such as FAI were also incorporated in some predictive models, as described in *Table 2*. After all imaging-based and tabular features were extracted, various feature selection methods including L1 and RFE and related packages on R and Python Sckitlearn, were utilized to choose optimal features for a model development. A summary of the imaging details and feature extraction and selection process is provided in Supplementary material online, *Tables S2*, and *S3*.

**Table 2:** Summary of main technical characteristics of predictive models

Author	AI algorithm used	Feature selection method	Type of input features	Training dataset					Test Dataset				
				AUC	95% CI	sensitivity	specificity	accuracy	AUC	95% CI	sensitivity	specificity	Accuracy
**Zhang *et al*. (2024)**	SVM, ML	LASSO	Imaging only	0.764	0.703–0.825	0.797	0.607	0.702	0.723	0.589–0.857	0.714	0.586	0.649
			Tabular only	0.752	0.689–0.815	0.46	0.946	0.702	0.706	0.564–0.847	0.464	0.862	0.667
			**Imaging** **+** **Tabular features**	**0.828**	**0.776–0.881**	**0.77**	**0.75**	**0.76**	**0.797**	**0.679–0.915**	**0.643**	**0.793**	**0.719**
**Miao *et al*.** ^34^	Random forest, ML	NA	Tabular only	0.838	0.777	0.841	0.857	0.847	NR				
	SVM, ML		Tabular + imaging features of the vessel	0.941	0.902–0.981	0.941	0.95	0.944	NR				
	SVM, ML		**Tabular** **+** **imaging features of both vessel and PVAT**	**0.949**	**0.913–0.986**	**0.9518**	**0.9508**	**0.9514**	NR				
**He *et al*.** ^35^	Ensemble ML model,	NA	**Imaging only**	**0.94**	**0.91–0.97**	**0.87**	**0.88**	**0.87**	**0.93**	**0.90–0.96**	**0.85**	**0.89**	**0.88**
**Chan *et al*.** ^8^	NR	NA	Tabular only	0·831	0.830–0.832	NR	NR	NR	NR				
			**Tabular** **+** **imaging features**	**0.854**	**0.851–0.857**	NR	NR	NR	NR				
**Militello *et al*.** ^36^	Random forest, ML	L1, tree based	Tabular only	0.684	0.596–0.772	0.642	0.621	0.628	NR				
		Tree based	Imaging only	0.819	0.745–0.893	0.767	0.681	0.720	NR				
		Mutual information	**Tabular** **+** **imaging features**	**0.820**	**0.744–0.896**	**0.770**	**0.716**	**0.739**	NR				
**You *et al*.** ^37^	ML	GBDT	Only imaging features from PVAT	0.69	0.616–0.763	0.604	0.682	0.69	0.703	0.59–0.81	0.604	0.68	0.64
			Only imaging features from EAT	0.543	0.46–0.62	0.446	0.56	0.482	0.543	0.41–0.66	0.446	0.56	0.482
			Tabular only	0.75	0.68–0.81	0.713	0.636	0.68	0.74	0.64–0.85	0.713	0.636	0.644
			**Tabular** **+** **imaging features from PVAT**	**0.782**	**0.71–0.84)**	**0.683**	**0.74**	**0.69**	**0.747**	**0.68–0.87**	**0.683**	**0.682**	**0.69**
**Wei He *et al*.** ^38^	XGBoost, ML	NA	Imaging features from plaque characteristics	0.92	0.88–0.96	0.86	0.85	0.85	0.84	0.68–0.99	0.83	0.82	0.73
			**Imaging features of both plaque characteristics and FAI**	**0.99**	**0.98–1.00**	**0.97**	**0.93**	**0.95**	**0.94**	**0.88–1.00**	**0.92**	**0.89**	**0.85**
**Oikonomou *et al*.** ^29^	Random forest, ML	RFE	**Imaging only (radiomics)**	NR					**0.774**	**0.62–0.92**	NA	NA	NA
**Huang *et al*.** ^28^	CNN, DL	NA	**Imaging only (radiomics)**	**0.969**	**0.938–1**	**0.907**	**0.92**	**0.913**	NR				
**Zhan *et al*.** ^39^	Random forest, ML	LASSO	Tabular only	0.81	0.74–0.88	0.74	0.7	0.71	0.67	0.53–0.81	0.67	0.62	0.63
			Imaging (FAI)	0.71	0.61–0.81	0.51	0.77	0.71	0.54	0.35–0.73	0.25	0.83	0.74
			**Imaging (PVAT Radiomics)**	**0.83**	**0.75–0.91**	**0.63**	**0.86**	**0.81**	**0.71**	**0.54–0.87**	**0.42**	**0.87**	**0.79**
**Huang *et al*.** ^40^	ML	NA	Tabular only	NA					0.84	NA	0.65	0.922	0.897
			Imaging (manual PVAT characteristic)	NA					0.817	NA	0.55	0.922	0.825
			Imaging (radiomics)	NA					0.961	NA	0.75	0.961	0.833
			**Tabular** **+** **Imaging features**	NA					**0.966**	NA	**0.85**	**0.98**	**0.944**

SVM, support vector machine; ML, machine learning; LASSO, least absolute shrinkage and selection operator; AUC, area under the curve; GBDT, gradient boosted decision tree; RFE, recursive feature elimination; FAI, fat attenuation index; PVAT, perivascular adipose tissue; EAT, epicardial adipose tissue; NA, not attainable; NR: not related.

##### Cardiovascular outcomes

The primary outcome in most of the included studies was the occurrence of MACE, which had a varied definition among studies but generally included myocardial infarction (MI), stroke, and cardiovascular mortality. Also, four studies^28,34,36,38^ only evaluated development of CAD, with one focusing on ACS events^28^ and another on early revascularization interventions in patients referred for CCTA.^38^

### Predictive performance of models

*One* of the studies used a deep learning convolutional neural network (CNN),^40^ and others utilized ML models for their final prediction model construction. *One* study did not explicitly report whether it had used ML- or DL-based models.^8^ From those utilizing ML, *Three* of the studies used a tree-based algorithm,^29,38,39^  *One* study developed a final ensemble ML model using multiple ML algorithms,^35^ and *three* studies compared the efficacy of different ML models.^25,34,36^  *Two* remaining studies did not mention the ML models they used.^37,40^ Considering performance metrics, including AUC, sensitivity, specificity, and accuracy, the best models suggested by different studies are summarized in *Table 2*, and a more comprehensive description of all conducted models can be found in Supplementary material online, *Table S4*. Most studies assessed model performance using internal resampling strategies during model development, most commonly cross-validation, reporting discrimination and classification metrics (e.g. AUC, sensitivity, and specificity). Furthermore, *three* studies also used external validation on separate cohort studies.^29,39,40^ Beside performance metrics, some studies reported their decision curve analysis as illustrated in Supplementary material online, *Table S3*.

Chan *et al*. conducted a multicentre longitudinal study in the UK to evaluate an AI-assisted MACE and cardiac mortality classification model based on FAI of pericoronary adipose tissue.^8^ Although the exact AI model was not mentioned in this study, it was reported that this classification was well correlated with both MACE and cardiac mortality (respectively, 4·68 [3·93–5·57] and 6·75 [5·17–8·82]). The other studies used either ML or DL methods, as summarized below.

#### Machine learning models

Although not reported the exact ML method used for modelling, You *et al*. used this method in their study evaluating the efficacy of a radiomics-based model either trained by PVAT or epicardial fat to predict MACE incidence within a 3-year follow-up after CCTA.^37^ This study showed that the PVAT-trained model has a higher AUC (0.703) than the epicardial fat-trained model (0.538). Incorporating PVAT radiomics with the clinical features of patients further enhanced the model's AUC to 0.781. Huang *et al*. designed a predictive MACE nomogram based on 12 PVAT-derived radiomics features.^40^ This nomogram, which integrated all clinical, radiomics, and conventional radiologic features into a single model, had an AUC of 0.97, which was higher than the AUCs of each clinical, radiologic, and radiomics models separately.

Three studies utilized tree-based models, two of which used random forest. In a case–control study, Oikonomou *et al*. developed a novel radiomic-based prognostic factor named FRP.^29^ After training and validating this factor using the random forest method, this model was further tested on the SCOT-HEART trial's eligible participants, which showed a better performance than traditional and previous CCTA-based calcium score, stenosis, and high-risk plaque stratification methods. In another study, Zhan *et al*. used random forest to compile a predictive model for MACE in patients with angina pectoralis-indicated CCTA within a one-year follow-up.^39^ Clinical, FAI, and radiomics models’ efficacy was then compared, which revealed higher performance metrics as well as net clinical beneficence for the radiomics model compared to the other models. All three models showed good agreement rates between observed and predicted incidence rates according to calibration curve. He *et al*. also used the XGBoost method to build a model predicting the need for PCI revascularization in patients referred for further assessment by CCTA to a tertiary hospital in China.^38^ The findings of this study revealed that adding FAI—as a feature of PCAT—to plaque characteristics could improve the AUC of the model from 0.84 to 0.95.

Some studies compared the accuracy of several ML models. Militello *et al*. used ML both in feature selection and model development.^36^ PVAT radiomics features from CCTA were selected by LASSO, tree-based, or mutual information. The final selected features were then fed to SVM, AdaBoost, XGBoost, or random forest to build a predictive model for CAD. The best final model was constructed by mutual information selection method followed by a random forest model with an AUC of 0.820 when incorporated with clinical data. When used separately, radiomics revealed a higher AUC than the clinical model (0.720 vs. 0.628). In contrast to this comparison, two other studies with relatively similar sample sizes reported that the SVM method had better performance metrics than tree-based models. In a study on 479 diabetic patients by Miao *et al*., adding 190 selected PVAT radiomics features to predictive models for CAD occurrence resulted in an AUC of 0.949 (0.913–0.986), which was better compared to AUCs of both clinical (0.804) and vessel radiomics plus clinical (0.941) models.^34^ Unlike most studies that compiled their model based on PVAT features derived from all three major coronary artery branches, Zhang *et al*. designed extra models for 4-year MACE prediction trained by features extracted from adipose tissue around each of the main coronary vessels. The final model trained by radiomics features of PVAT around all three arteries showed the highest AUC of 0.723.^25^ When this radiomics model was added to the clinical model, its performance was further increased to AUC 0.797. Regarding clinical validity, findings of this study revealed an excellent overall net benefit across most probability thresholds and excellent calibration rate.

Regarding these variances in the efficacy of different ML models for cardiovascular prediction, He *et al*. used an ensemble ML model by combining different models such as GNB, LDA, SVM, and AdaBoost for MACE prediction.^35^ While the Framingham score had an AUC of 0.62 and the conventional logistic regression model had an AUC of 0.89, the ensemble model showed an AUC of 0.94 (0.91–0.97) in the training and 0.93 (0.90–0.97) in the validation cohort. Among different ML models used in this study, ML AdaBoost and SVM algorithms showed the highest AUC, respectively, across training and test sets, as noted in Supplementary material online, *Table S4*, however, Decision curve analysis (DCA) showed the highest clinical net benefit for ensemble model.

#### Deep learning models

Apart from ML models, Huang *et al*. proposed a CNN-based deep learning model named TSCFE-CFF in 2023.^28^ This model was able to extract the most correlated CCTA features according to both PCAT and plaque features and simultaneously build an ACS predictive model that showed enhanced performance metrics compared to all previous state-of-the-art medical image classification methods (AUC=0.965).

### Meta-analysis findings

Based on 11 studies and more than 81 predictive models, pooled AUCs were synthesized using inverse-variance random-effects meta-analysis (DerSimonian–Laird) across extracted model evaluations (i.e. unique algorithm–feature set–dataset split combinations), with standard errors derived from reported 95% confidence intervals, and the cumulative results yielded in a pooled AUC estimate of 87.9% (95% CI: 83.2%–92.6%) with a prediction interval of 68.9% to 100% for the best models and a pooled AUC estimate of 82.2% (95% CI: 81.0%–83.3%) with a prediction interval of 74.6% to 89.7%. The *Z*-value tests rejected the null hypothesis (Z-value of 36.376 and 140.395, respectively, with *P* < 0.001, and using a criterion alpha of 0.050). Heterogeneity assessment for the best models confirmed a substantial and statistically significant heterogeneity (*I*^2^ = 98.326%, *Q* = 776.631, *P* < 0.001). Similar results were obtained from the analysis of all models (*I*^2^ = 96.355%, *Q* = 2055.774, *P* < 0.001).

Subgroup analysis of the best models revealed a not statistically significant difference based on the AI subtype type that was used to develop the predictive models (*P* = 0.061); however, the subgroup analysis of all included models resulted in a significant difference, favoring DL models [86.6% (95% CI: 81.6%–91.6%), with a prediction interval of 74.8% to 98.4%]. more than ensemble models [85.2% (95% CI: 80.9%–89.4%), with a prediction interval of 73.7% to 96.6%], and to a lesser degree ML models [81.7% (95% CI: 79.7%–83.6%), with a prediction interval of 70.8% to 92.5%], while all these models significantly exceeded LR [76.4% (95% CI: 73.5%–79.4%), with a prediction interval of 65.4% to 87.5%] predictive models (*P* < 0.001).

Subgrouping based on the features that were used to develop the models showed to pose a significant impact on the final AUC, as in the analysis of all models that were shown to be in favour of the models that were based on clinical (only tabular features) [76.3% (95% CI: 72.7%–80.0%), with a prediction interval of 64.9% to 87.8%], radiomics (only imaging features) [78.2% (95% CI: 75.7%–80.6%), with a prediction interval of 67.0%–89.3%], and combined (multimodal features) models [85.0% (95% CI: 82.9%–87.2%), with a prediction interval of 74.0%–96.1%], respectively (*P* < 0.001). However, these significances were n't observed in the best models subgroup analysis (*P* = 0.059).

A significant trend to higher AUC was also observed in the group with 10-fold validation features [80.1% (95% CI: 78.1%–82.1%), with a prediction interval of 72.4%–87.8%] compared to fivefold validation features [81.5% (95% CI: 80.0%–83.1%), with a prediction interval of 74.0%–89.1%] (*P* < 0.001) in the analysis of all models; however, the results for the best models were inconclusive and not statistically significant (*P* = 0.703).

Notably, no significant differences were observed comparing single-centre and multicentre studies, neither in the analysis of the best models nor in all models (*P* = 0.288 and *P* = 0.603, respectively).

Study design also showed to cause significant differences in the subgroup analysis of all models, with a tendency to have a higher AUC in prospective studies [85.4% (95% CI: 83.6%–87.1%), with a prediction interval of 78.8%–91.9%], and on lesser degrees in retrospective cohort [83.0% (95% CI: 81.5%–84.4%), with a prediction interval of 76.5%–89.4%], and case–control studies [73.0% (95% CI: 69.9%–76.1%), with a prediction interval of 65.9%–80.0%], while none was observed in the analysis of the best models (*P* = 0.192).

Meta-regression analysis identified the influence of the following moderators: male gender (β = 0.5459, *P* = 0.001), age (β = 0.0211, *P* < 0.001), study population (β < 0.001, *P* < 0.001), and patients BMI (β = −0.0759, *P* < 0.001) on the AUC.

**Figure:**
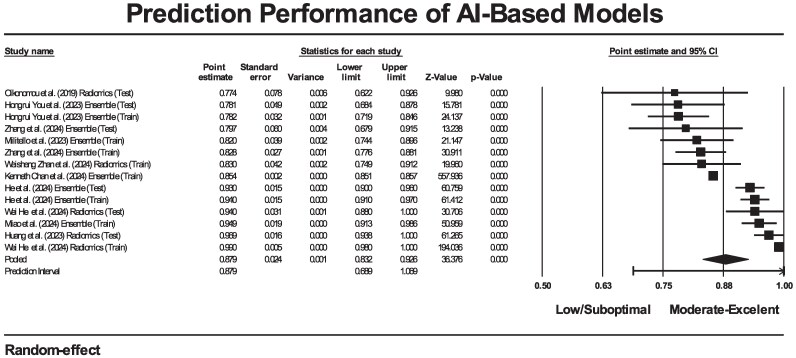


**Figure:**
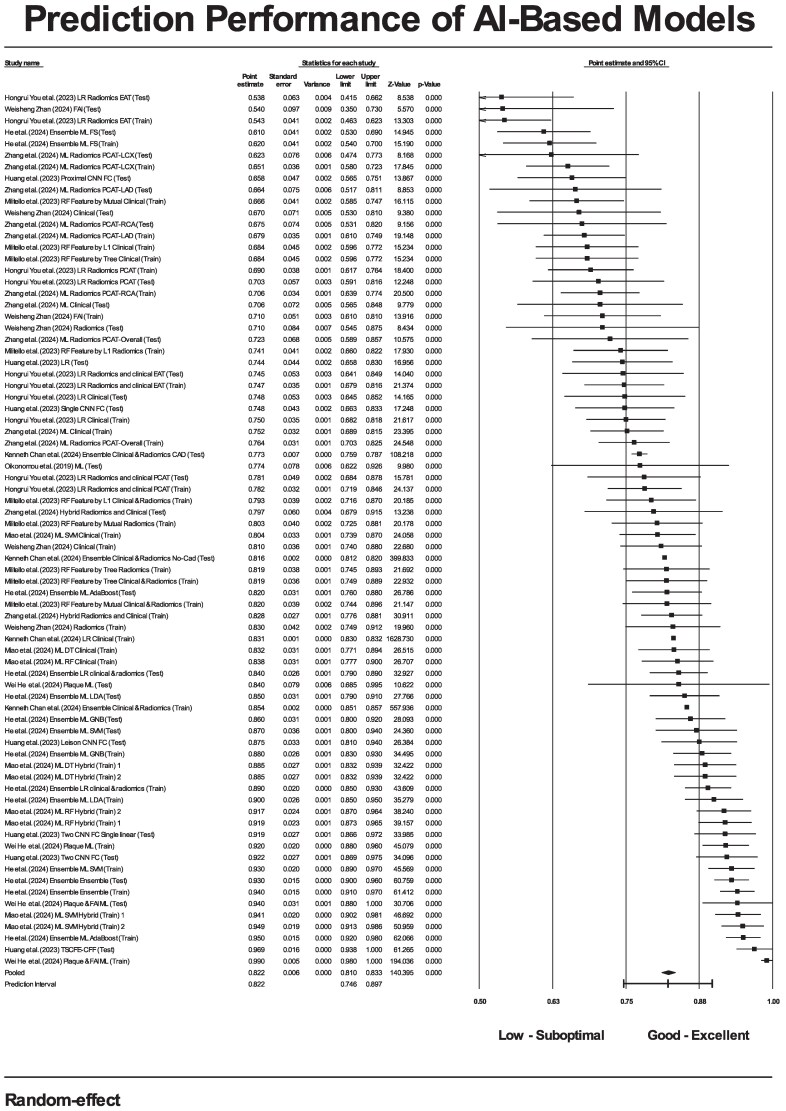


#### Sensitivity analysis of all AI-based models

Across all 74 model evaluations (*k* = 74 AUC effect sizes eligible for pooling; each evaluation corresponds to one reported model-performance estimate in either a development/training (including cross-validation) cohort or a validation/test cohort across the included studies), the random-effects meta-analysis yielded a pooled AUC of 0.819 (95% CI: 0.806–0.832), indicating good to excellent predictive performance for major adverse cardiovascular events (MACE) using CCTA-derived adipose tissue characteristics. Between-study heterogeneity was substantial (*I*^2^ = 96.2%, τ^2^ = 0.001 88, *Q* = 1902.0, *P* < 0.001), with a 95% prediction interval of 0.733–0.905, confirming the persistence of variability but within a clinically meaningful range of effect sizes.

'The leave-one–effect-size-out (leave-one model evaluation out) influence analysis revealed minimal impact of any single model evaluation on the pooled results. The recalculated pooled AUCs across LOO iterations ranged from 0.802 to 0.824, and heterogeneity indices remained high (*I*^2^ = 92.6%–96.2%) but stable, indicating that no single study materially altered the pooled performance or the overall heterogeneity profile.

The most influential omissions were Chan *et al*. (Ensemble Clinical & Radiomics, Train; pooled AUC 0.802, ΔAUC −0.017) and Chan *et al*. (Logistic Regression Clinical, Train; pooled AUC 0.803, ΔAUC −0.015). However, even these influential points did not substantially modify the global interpretation. These findings demonstrate that the observed heterogeneity is structural rather than artifactual, reflecting diversity in imaging feature sets (e.g. perivascular fat attenuation index [FAI], hybrid plaque–radiomic features), AI frameworks (logistic regression, ensemble, gradient boosting, CNN-based architectures), and endpoint definitions, rather than the presence of statistical outliers or model instability. Therefore, despite the imbalance in total participant contribution across studies, no single study/model materially dominated the pooled AUC estimate, as supported by the narrow LOO AUC range (0.802–0.824) and small maximum ΔAUC.

#### Sensitivity analysis of best-performing models

When analysis was restricted to the best-performing model per study (*n* = 51; test set preferred), the pooled discriminative performance improved to AUC = 0.836 (95% CI: 0.824–0.848), confirming robust and consistent prognostic accuracy across the top-tier AI models. Despite the refined dataset, heterogeneity remained high (*I*^2^ = 92.0%, τ^2^ = 0.000 68, *Q* = 625.15), again representing true diversity in methodological design and imaging pipelines. The 95% prediction interval (0.784–0.888) indicated that even the lower-bound estimates of model performance retained clinical utility.

The leave-one-study-out (LOO) influence analysis for this subset (one selected model evaluation per study) confirmed high robustness of the pooled estimate, with recalculated AUCs ranging from 0.809 to 0.840 and *I*^2^ varying modestly between 87.3% and 92.2%.

The largest deviation occurred when the Kenneth Chan *et al*. Ensemble Clinical & Radiomics model was omitted (pooled AUC 0.809, ΔAUC −0.027), yet the overall classification accuracy remained excellent. Other influential omissions—such as He *et al*. (Ensemble ML-FS, Test) and Huang *et al*. (TSCFE-CFF, Test)—produced negligible shifts (ΔAUC < 0.005), underscoring the stability of the meta-analytic estimate.

**Table:** 

Dataset	*k* (models)	Pooled AUC	95% CI	*I* ^2^	τ^2^	95% PI	LOO AUC Range	LOO *I*^2^ Range
**All Models**	74	0.819	0.806–0.832	96.2%	0.001 88	0.733–0.905	0.802–0.824	92.6%–96.2%
**Best Models**	14	0.836	0.824–0.848	92.0%	0.000 68	0.784–0.888	0.809–0.840	87.3%–92.2%

**Figure:**
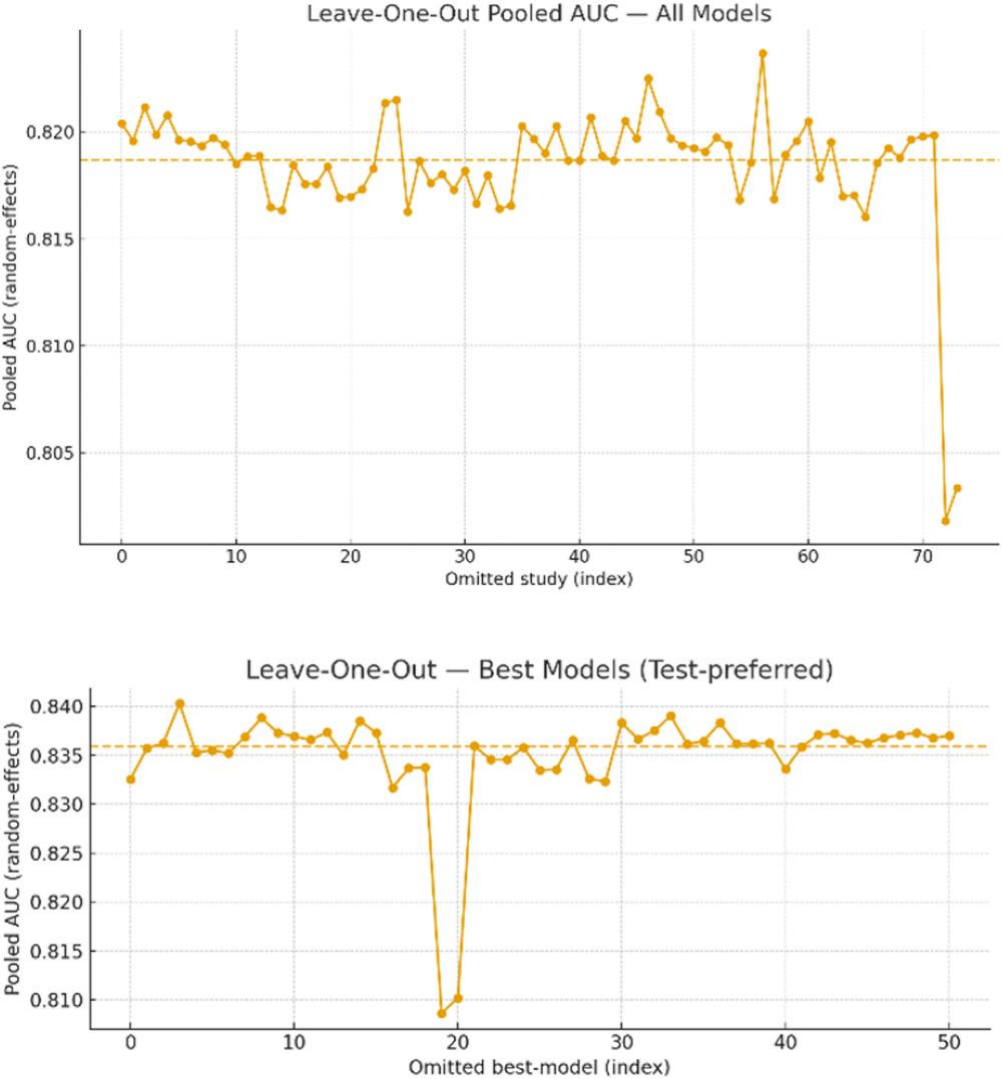


#### Publication Bias

Egger's regression test demonstrated no significant evidence of publication bias in the analysis of the best models (B₀ = 2.5494, 95% CI: −2.6065 to 7.7053; *t* = 1.0773; *P* = 0.1513 [one-tailed], *P* = 0.3025 [two-tailed]). Similarly, in the analysis of all models, Egger's test confirmed the absence of significant publication bias (B₀ = −0.028 47, 95% CI: −1.290 16 to 1.233 22; t = 0.044 96; *P* = 0.482 13 [one-tailed], *P* = 0.964 26 [two-tailed]).

## Discussion

This systematic review identified 11 studies involving 47 244 participants from Asia, the United States, and Europe that examined AI-based predictive models for MACE. Most of these studies utilized adipose tissue segmentations from CCTA and developed predictive models that incorporated radiomic, clinical, and conventional imaging features of PVAT. The meta-analysis demonstrated that radiomics-based models, particularly those integrating perivascular FAI and epicardial adipose tissue (EAT) characteristics, achieved superior performance metrics. Pooled AUCs were 85.0% (95% CI: 82.9%–87.2%) for combined models and 78.2% (95% CI: 75.7–80.6%) for radiomics alone, significantly outperforming classical non-radiomic models (AUC 76.3%, 95% CI: 72.7%–80.0%, *P* < 0.001). Importantly, AI-based models consistently showed higher accuracy than traditional clinical risk scores, such as QRISK3 and the Framingham score, in predicting MACE and enhancing risk stratification. These findings highlight the ability of AI approaches to process large, multidimensional datasets and capture high-level imaging biomarkers that are not accessible through conventional methods. Although the pooled AUC was 87.9% (95% CI: 83.2$–92.6%), the observed heterogeneity across studies (*I*^2^ = 98.326%, *P* < 0.001) underscores the need for standardized methodologies in model development and reporting.

Cardiovascular disease remains a leading cause of morbidity and mortality worldwide, necessitating accurate and timely risk stratification. Traditional models such as the Framingham Risk Score, QRISK3, coronary calcium scoring, and models based on invasive coronary angiography have provided valuable prognostic information but are constrained by time demands, complexity, and error susceptibility, which may compromise patient outcomes. AI-based models address these shortcomings by leveraging computational power to analyze complex, high-dimensional imaging data and incorporating novel biomarkers such as perivascular FAI and EAT features.^41,42^ These approaches align with broader advances in predictive cardiology, where features like FAI are increasingly studied for their prognostic value.^43^ Nevertheless, conventional methods remain limited. For example, a meta-analysis by Bell *et al*. found no additional clinical benefit when coronary artery calcium scores (CACSs) were incorporated into traditional risk assessment models.^44^

Similarly, while FAI reflects localized coronary inflammation and can predict complications such as in-stent restenosis,^45^ its utility in long-term outcome prediction is limited. Some studies, such as that by Oikonomou *et al*., have addressed these limitations by combining fat radiomic profiles features with ML-based predictive models, which capture persistent PVAT remodelling and improve both primary and secondary prevention.^29^ Subgroup analyses of our study reinforced the superiority of AI-based models according to statistical performance metrics. Deep learning (DL) approaches achieved the highest pooled AUC (86.6%, 95% CI: 81.6%–91.6%), followed by ensemble models (85.2%, 95% CI: 80.9%–89.4%) and ML models (81.7%, 95% CI: 79.7%–83.6%), while classical logistic regression–based models remained lower (76.4%, 95% CI: 73.5%–79.4%).

A basic mechanistic understanding of different AI algorithms may help clinicians critically appraise performance metrics and incorporate them into decision-making. ML models, with a pooled AUC of 81.7%, and particularly ensemble tree-based methods such as RF, Extreme Gradient Boosting (XGB), and Gradient Boosting (GB), proved robust for structured data and smaller sample sizes. These approaches highlight feature importance, reduce overfitting, and handle imbalanced data at lower computational costs, especially when combined with radiomics and clinical features. Ren *et al*. reported superior performance of an RF model integrating clinical variables and imaging-based score compared with logistic regression.^46^ Other ML models, such as SVM, showed strong classification performance in high-dimensional data but require larger datasets and higher computational resources.^47^ In the PACIFIC trial, Lin *et al*. showed that an ML model integrating CCTA-based plaque features accurately predicted ischemia detected by fractional flow reserve modality and impaired myocardial blood flow by PET, outperforming standard CCTA stenosis evaluation and performing comparably to fractional flow reserve CT.^48^ More recently, Pezel *et al*. developed a multimodal ML model using CCTA and MRI data, achieving an AUC of 86% for predicting MACE, significantly outperforming traditional risk scores and imaging-based assessments.^49^

DL approaches, particularly CNNs, achieved the highest predictive performance (pooled AUC 86.6%, 95% CI: 81.6%–91.6%). These models are particularly effective in analyzing unstructured imaging data, integrating segmentation, feature extraction, and prediction within a single pipeline. Their ability to capture complex imaging patterns enhances predictive accuracy, as demonstrated in studies achieving AUCs above 0.96.^28,50^ DL-based algorithms have also been shown to perform non-inferior to expert readers in detecting coronary stenosis, with higher sensitivity and negative predictive value.^51^ Unlike ML models, CNNs bypass manual feature engineering, reducing operator bias and improving reproducibility. Nevertheless, when comparing performance metrics of DL to ML methods, we should also consider the fact that these models mostly are trained by richer and more complex features. Furthermore, the study which utilized DL algorithm in this review, mentioned no external validation method as opposed to many ML-based records. Therefore a general conclusion for the efficiency of different AI categories which only rely on performance metrics like AUC might still be inaccurate and confounded by factors like input complexity and validation strategy.

Ensemble approaches, with a pooled AUC of 85.2% (95% CI: 80.9%–89.4%), demonstrated lower variance and reduced overfitting compared to standalone models by combining predictions from multiple models. These models proved to be extremely useful, particularly when sample sizes are small, and data usage needs to be optimized to enhance generalizability. They required less extensive validation compared to deep learning frameworks, offering a practical solution for resource-constrained settings. As a result, ensemble models exhibited the smallest gaps between training and validation performance among all of the models in our study.

Our findings highlight several clinically relevant aspects. **First**, AI-driven CCTA analysis can refine risk stratification, particularly in asymptomatic patients, in whom up to two-thirds of MACE occur without prior symptoms. By integrating adipose tissue biomarkers with plaque and clinical features, AI models provide individualized risk estimates that may guide preventive strategies and optimize the use of invasive testing or therapies. **Furthermore**, these models may improve efficiency in clinical workflows. While traditional scoring systems are limited by subjectivity and operator variability, AI can automate segmentation, feature extraction, and prediction, reducing time burden and potentially lowering costs.

Despite promising results, our review identified several key challenges limiting the clinical implementation of these findings. First, substantial methodological variability contributed to marked heterogeneity (*I*^2^ > 96%, *P* < 0.05). Differences in adipose tissue segmentation approaches (manual, semi-automated, or automated tools such as Pyradiomics or EATseg), inconsistent MACE definitions, and variation in follow-up duration limited comparability across studies.^52,53^ Reporting of model architecture, feature selection procedures, and validation strategies was frequently incomplete, restricting reproducibility. Adoption of standardized reporting frameworks such as TRIPOD-AI^54^ and CONSORT-AI,^55^ is therefore essential.

Second, several sources of bias may have inflated reported AUC values. The main area which imposed high risk of bias on some of our included studies was analytical domain. Particularly with radiomics studies and other models relying on high-dimensional features, working on datasets with a reasonable incidence rate of our main outcome is of high importance. In studies with low incidence of the main outcome, complex models developed using numerous features have a higher risk of overfitting.^28,34–36,39^ Therefore, working on large multicentre datasets is essential to decrease class imbalance and variance of researchers’ work. Another issue increasing the risk of bias for many included records was a lack of external validation that may raise serious concerns on generalizability of the performance metrics in real life settings.^25,28,35–39^ Although cross-validation reduces variance in performance estimation, if feature selection or other preprocessing steps are applied outside the validation loop, this can lead to substantial positive bias in AUC estimates, as shown in radiomics studies where incorrect cross-validation workflows introduced AUC increases of up to 0.15.^56^ than restricting them to training folds, can lead to even larger performance inflation, with reported AUC increases of up to 0.34 in imbalanced datasets^57^The other methodological flaw noted in some of the included papers in this study was using univariable analysis instead of more robust methods like LASSO for feature selection.^34,36,38,39^ It is known that, especially in health research many variables may jointly predict occurrence of outcomes so utilizing univariable methods like *P* value extraction, etc. might lead to a bias in coefficient estimate by ignoring interaction between different features. Apart from these analytical problems, one other source of bias among included studies was the bias associated with participant domain.^28,34–36,38,39^ Alongside insufficient missing data handling strategies, another point regarding predictive efficacy of AI-powered algorithms based on imaging data is the quality of imaging. Lack of predefined standards for quality control as well as reasonable protocols on whether to include records with low quality imaging data or not, creates subjective differences that make pooling the final results of different studies together challenging. Furthermore, improper application of oversampling techniques before cross-validation, rather. These findings underscore the susceptibility of high-dimensional radiomic models to data leakage and overly optimistic discrimination estimates.

**Third, interpretability and transparency** were insufficiently addressed. Few studies incorporated explainable AI (XAI) tools, such as SHAP or LIME, to clarify prediction drivers.^58^ This lack of interpretability may hinder clinical trust and implementation. **Fourth**, most studies primarily reported discrimination metrics such as AUC. Although AUC quantifies a model's ability to distinguish between patients who will and will not experience events, it does not evaluate calibration, that is, the agreement between predicted and observed risk, which is equally critical in cardiovascular risk prediction.^59,60^ Moreover, discrimination alone does not ensure clinical usefulness, as treatment decisions rely on predefined risk thresholds rather than global ranking performance. Accordingly, comprehensive evaluation of prediction models should extend beyond discrimination statistics to include calibration assessment and clinical utility measures.

**Fifth**, concerning the interpretation of heterogeneity and robustness, although heterogeneity remained high in all scenarios, this reflects the intrinsic diversity of AI architectures, feature extraction methods, outcome definitions, follow-up durations, and study design variability (single-centre vs. multicentre, retrospective vs. prospective cohorts). Such diversity represents real-world variability in model development and validation strategies rather than analytical inconsistency. Importantly, the robustness of the pooled AUC estimate under all sensitivity scenarios supports the conclusion that AI models leveraging adipose tissue–derived features from CCTA consistently achieve strong predictive performance for MACE, irrespective of model subtype or dataset origin. Finally, the concentration of studies in specific geographic regions limits broader applicability.

Future work should prioritize large, prospective, multicentre studies with standardized endpoints and transparent reporting. Integration of AI models into electronic health records and imaging platforms will be critical for seamless clinical adoption. Cost-effectiveness analyses are needed to determine whether AI-guided strategies improve outcomes without disproportionate resource use. Finally, regulatory frameworks, data governance, and ethical considerations must be addressed to ensure safe, equitable implementation of AI in cardiovascular risk prediction.

## Conclusion

AI-based predictive models integrating CCTA-derived adipose tissue biomarkers demonstrate promising discriminative performance for MACE risk stratification, particularly when combining FAI, EAT, plaque characteristics, and clinical variables. However, the predominance of internal validation, substantial methodological heterogeneity, and limited use of independent external cohort temper conclusions regarding immediate clinical readiness. Although pooled AUC estimates suggest improved discrimination compared with traditional risk scores, calibration, transportability, and real-world clinical utility remain insufficiently established. Rigorous external validation in prospective, multicentre settings, alongside standardized reporting and transparent modelling workflows, will be necessary to determine whether these models can achieve reliable performance beyond controlled research environments

## Supplementary Material

ztag104_Supplementary_Data

## Availability of data and material (data transparency)

The data underlying this article will be shared on reasonable request to the corresponding author.
