# Supplementary material for: Role of artificial intelligence in developing predictive models for major adverse cardiovascular outcomes using CCTA adipose tissue characteristics: a systematic review and meta-analysis
Source: Eur Heart J Digit Health. 2026 Jul 14;7(6):ztag104. doi: 10.1093/ehjdh/ztag104 (PMC13367595; doi:10.1093/ehjdh/ztag104)
Supplement: ztag104_Supplementary_Data [file ztag104_supplementary_data.zip › Supplementary for FIGURES.docx]

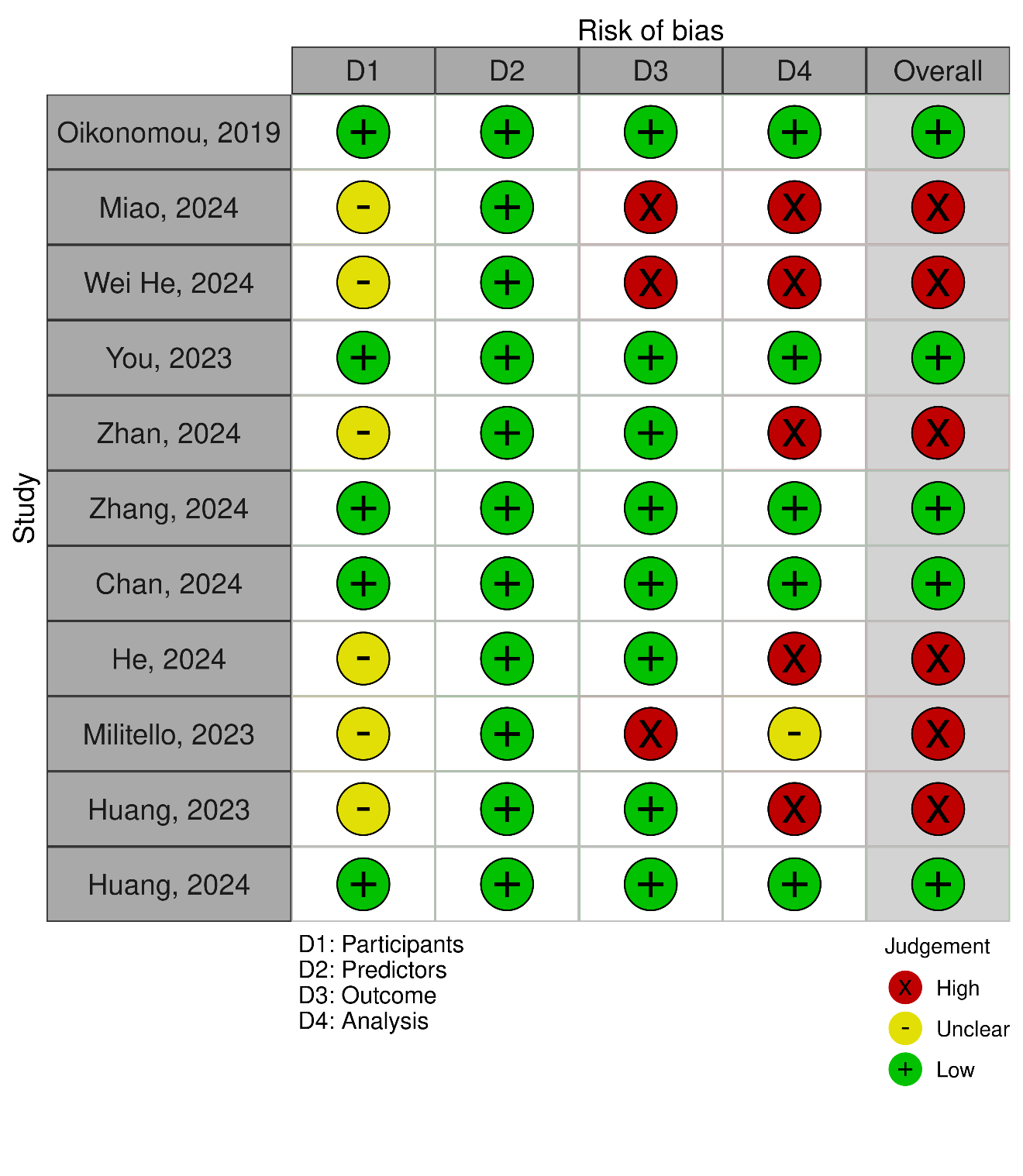

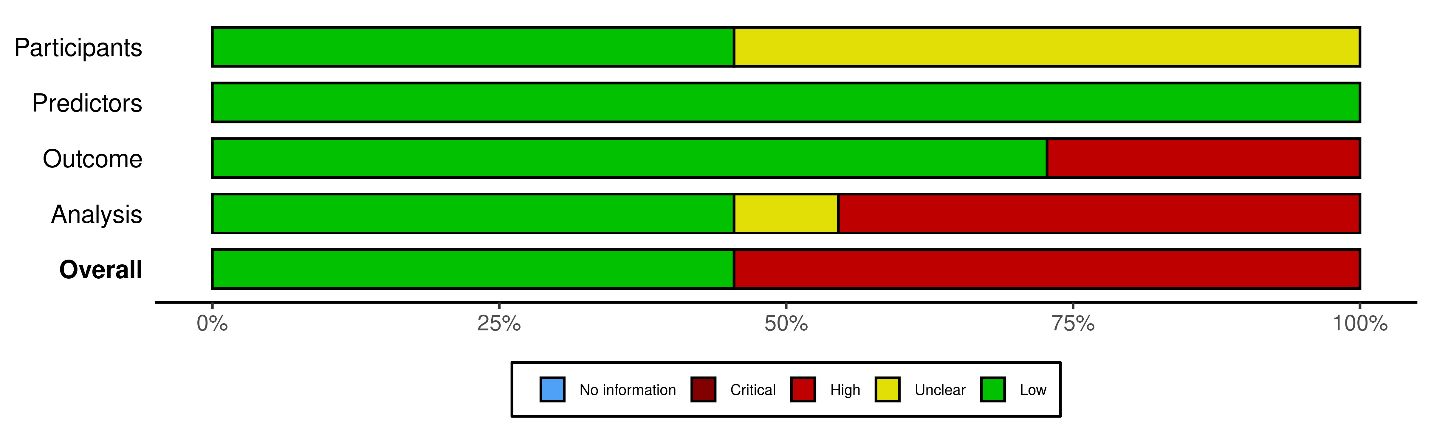


**Figures 1, and 2**
*Supplementary Illustration for risk of bias*

Records identified from:

Databases (n = 1584)

Pubmed (n = 231)

Scopus (n = 354)

Web of science (n = 219)

Embase (n = 622)

Google Scholar (n = 158)

Records removed *before screening*:

Duplicate records removed by Endnote screening (n = 524)

Duplicate records removed by Rayyan Software (n = 186)

**Identification**

**Included**

Reports sought for retrieval

(n = 172)

Reports not retrieved

(n = 1)

Full text of Reports assessed for eligibility

(n = 171)

Reports excluded:

Wrong Publication Type (n = 64)

Not CCTA (n = 22)

Not AI (n = 10)

Not MACE (n = 35)

Not adipose tissue (n = 12)

Not English (n = 4)

Only used AI for imaging (n=13)

Studies included in review

(n = 11)

Articles screened by Title/Abstract

(n = 874)

Articles excluded by Title/Abstract

(n = 702)

**Screening**

**Identification of studies via databases and registers**

*Consider, if feasible to do so, reporting the number of records identified from each database or register searched (rather than the total number across all databases/registers).

**If automation tools were used, indicate how many records were excluded by a human and how many were excluded by automation tools.

**Figure 3**
*PRISMA chart of included studies*

*Consider, if feasible to do so, reporting the number of records identified from each database or register searched (rather than the total number across all databases/registers).

**If automation tools were used, indicate how many records were excluded by a human and how many were excluded by automation tools.

Source: Page MJ, et al. BMJ 2021;372:n71. doi: 10.1136/bmj.n71.

This work is licensed under CC BY 4.0. To view a copy of this license, visit <https://creativecommons.org/licenses/by/4.0/>
