# Supplementary material for: Role of artificial intelligence in developing predictive models for major adverse cardiovascular outcomes using CCTA adipose tissue characteristics: a systematic review and meta-analysis
Source: Eur Heart J Digit Health. 2026 Jul 14;7(6):ztag104. doi: 10.1093/ehjdh/ztag104 (PMC13367595; doi:10.1093/ehjdh/ztag104)
Supplement: ztag104_Supplementary_Data [file ztag104_supplementary_data.zip › Supplementry files revised.docx]

Supplementary Table 1: summary of search strategy in different databases

|  | **Search string** | results |
| --- | --- | --- |
| **PubMed** | ((((((((((Perivascular adipose[Title/Abstract]) OR (Perivascular fat[Title/Abstract])) OR (PVAT[Title/Abstract])) OR (Fat[Title/Abstract])) OR (Adipose[Title/Abstract])) OR (Vascular fat[Title/Abstract])) OR (Vascular adipose[Title/Abstract])) OR (perivascular[Title/Abstract])) AND ((((((((((((((((((((((((Cardiovascular diseases[MeSH Terms]) OR (Coronary artery disease[MeSH Terms])) OR (Stroke[Title/Abstract])) OR (Coronary*[Title/Abstract])) OR (Cardiovascular[Title/Abstract])) OR (Infarction[Title/Abstract])) OR (Ischemia[Title/Abstract])) OR (Aort*[Title/Abstract])) OR (Heart[Title/Abstract])) OR (Angina[Title/Abstract])) OR (Cardiac[Title/Abstract])) OR (Cerebrovascular[Title/Abstract])) OR (Brain[Title/Abstract])) OR (Artery[Title/Abstract])) OR (Scleros*[Title/Abstract])) OR  (Aneurysm*[Title/Abstract])) OR (Ventric*[Title/Abstract])) OR (Atri*[Title/Abstract])) OR (MI[Title/Abstract])) OR (Thrombos*[Title/Abstract])) OR (Stent[Title/Abstract])) OR (Peripher*[Title/Abstract])) OR (Vascular[Title/Abstract])) OR (Athero*[Title/Abstract]))) AND (((((((Computed tomography[Title/Abstract]) OR (CCTA[Title/Abstract])) OR (CTCA[Title/Abstract])) OR (Angiograph*[Title/Abstract])) OR (Coronary computed tomography angiography[Title/Abstract])) OR (Coronary CT[Title/Abstract])) OR (Coronary computed[Title/Abstract]))) AND ((((((((Artificial intelligence[MeSH Terms]) OR (Artificial intelligence[Title/Abstract])) OR (machine learning[Title/Abstract])) OR (machine learning[MeSH Terms])) OR (Deep learning[Title/Abstract])) OR (AI[Title/Abstract])) OR (Neural network[Title/Abstract])) OR (Computer[Title/Abstract])) | 231 |
| **Scopus** | TITLE-ABS-KEY(("cardiovascular disease*" OR "coronary artery disease*" OR stroke OR heart OR coronary OR cardiovascular OR infarction OR ischemia OR aort* OR  angina* OR cardiac OR cerebrovascular OR brain OR artery OR sclerosis* OR athero* OR aneurysm* OR ventric* OR atri* OR MI OR thrombos* OR stent OR peripher* OR vascular)) AND TITLE-ABS-KEY(("machine learning" OR "deep learning" OR "artificial intelligence" OR AI OR "neural network")) AND TITLE-ABS-KEY(("computed tomography" OR CCTA OR CTCA OR "coronary computed tomography angiography" OR "coronary CT")) AND TITLE-ABS-KEY(("perivascular fat" OR "perivascular adipose" OR PVAT OR fat OR adipose OR "vascular fat" OR "vascular adipose")) | 354 |
| **Web of Science** | TS=("cardiovascular disease*" OR "coronary artery disease*" OR stroke OR heart OR coronary OR cardiovascular OR infarction OR ischemia OR aort* OR angina* OR  cardiac OR cerebrovascular OR brain OR artery OR sclerosis* OR athero* OR aneurysm* OR ventric* OR atri* OR MI OR thrombos* OR stent OR peripher* OR  vascular) AND TS=("machine learning" OR "deep learning" OR "artificial intelligence" OR AI OR "neural network") AND TS=("computed tomography" OR CCTA OR CTCA OR "coronary computed tomography angiography" OR "coronary CT") AND TS=("perivascular fat" OR "perivascular adipose" OR PVAT OR fat OR adipose  OR "vascular fat" OR "vascular adipose") | 219 |
| **Embase** | ('cardiovascular disease'/exp OR 'coronary artery disease'/exp OR 'cardiovascular disease*' OR 'coronary artery disease*' OR stroke OR heart OR coronary OR  cardiovascular OR infarction OR ischemia OR aort* OR angina* OR cardiac OR cerebrovascular OR brain OR artery OR sclerosis* OR athero* OR aneurysm* OR  ventric* OR atri* OR MI OR thrombos* OR stent OR peripher* OR vascular) AND ('artificial intelligence'/exp OR 'machine learning'/exp OR 'deep learning'/exp OR  'artificial intelligence' OR 'machine learning' OR 'deep learning') AND ('computed tomography'/exp OR CCTA OR CTCA OR 'coronary computed  tomography angiography' OR 'coronary CT') AND ('perivascular adipose tissue'/exp OR 'perivascular fat' OR 'perivascular adipose'  OR PVAT OR fat OR adipose OR 'vascular fat' OR 'vascular adipose') | 622 |
| **Google Scholar** | ("cardiovascular disease" OR "coronary artery disease" OR "cardiovascular outcome*" OR stroke OR MI OR angina OR thrombosis* OR cardiovascular OR atherosclerosis OR heart) AND ("machine learning" OR "artificial intelligence" OR "deep learning" OR AI) AND (CCTA OR CTCA OR "coronary CT" OR angiograph* OR "coronary computed tomography angiography") AND ("perivascular adipose tissue" OR "perivascular fat" OR "vascular fat" OR  "vascular adipose" OR fat OR adipose OR perivascular OR "vascular fat" OR "vascular Adipose") | 158 |

| **Table 2**  **Author** | **Scanner Details**  **(e.g., scanner type, resolution)** | **Scanner Parameters** | **Segmentation**  **method/software** | **Feature extraction software** |
| --- | --- | --- | --- | --- |
| Zhang et al. (2024) | 256 slice, Brilliance iCT, Philips Medical systems | collimation: 128*0.625 mm, rotation time: 270 ms, tube voltage: 100 or 120 kV (depending on BMI), tube current: 500-700 mA  0.6–0.8 ml/kg of iodinated contrast was injected (Ioversol 320mgI/ml) at a flow rate of 4–6 ml/s | Perivascular Fat Analysis Tool software( Shukun Technology Co., Ltd) | Perivascular Fat Analysis Tool software( Shukun Technology Co., Ltd) |
| Miao et al. (2024) | Siemens and GE computerized tomography instruments | NM | A weekly supervised method using centerline constraint | Pyradiomics opensource python package |
| He et al. (2024) | Siemens SOMATOM Definition AS (Siemens Medical Systems, Erlangen, Germany). | NM | manual adjustment on the basis of automatic segmentation confirmed by two expert radiologists | Deepwise Coronary Artery Analysis Software (Deepwise Inc., Beijing) |
| Chan et al. (2024) | NM | NM | NM | CaRi-Heart version 2.5 device (Caristo Diagnostics, Oxford, UK) :to generate the FAI Score for each coronary artery and the AI-Risk for the patient according to the quality standards regulating medical devices |
| Militello et al. (2023) | NM | NM | Segmentation technique: a semi-automated method developed in matlab | PyRadiomics |
| You et al. (2023) | 256-slice (Brilliance iCT, Philips Medical Systems, Cleveland, USA) | collimator 128 * 0.625 mm, tube voltage 100-120 kV, tube current automatically adjusted, layer spacing 0.45 mm, layer thickness 0.9 mm, and rotation time 270 ms.  Contrast agent (Iopromide 370; Bayer Schering, Berlin, Germany; 0.6 or 0.8 ml/kg) injected through the anterior elbow vein at a flow rate of 4.5-5.5 ml/s via a high-pressure injector + 40 ml flush of saline at the same rate | EATseg software for semi-automatic segmentation, manually adjusted on 3D slicer software by two radiologists. | NM |
| Wei He et al. (2024) | 320 row (Aquilion One Vision Canon Medical Systems Corporation) | Tube voltage =120 kV, collimation =320 × 0.5 mm, tube current = 500 mA, gantry rotation time =275 ms. , 70 mL of iodinated contrast (iopamidol, 370 mg iodine/mL, Bracco Sine Pharmaceutical Corp. Ltd) injected intravenously at a rate of 4.2 mL/s. | Syngo fastView, VX57I33; Siemens Healthineers) workstation. | Plaque characteristics were measured using the semi-automatic plaque analysis software (Coronary Plaque Analysis, version2.0, Siemens Healthineers) with manual corrwctions. |
| Huang et Al. (2023) | Either a 256-slice CT scanner or a dual-layer spectral detector CT | Resolution:0.45 mm x 0.45 mm x 0.625 mm | Manual | NM |
| Oikonomou et al. (2024) | Eitherr a 256-slice Brilliance iCT scanner (Philips Medical Systems, Best, The Netherlands), a 2 x 128-slice Definition Flash scanner (Siemens Healthcare, Erlangen, Germany) or a 2 x 192-slice Somatom Force CT scanner (Siemens Healthcare, Forchheim, Germany) | 0.35 sec rotation time, 2.5 mm axial slice thickness, 20 mm detector coverage, tube energy of 120 kV and 200 mA | Aquarius WorkstationVR V.4.4.11-13, TeraRecon Inc., Foster City, CA, USA for basic segmentation and CaRi-HEART proprietary algorithms (Caristo Diagnostics Ltd, Oxford, UK) for final calculation | 3D slicer for radiomics feature calculation, SlicerRadiomics |
| Zhan et al. (2024) | 2*96-detector row (Stellar Infinity detector, SOMATOM Force, Siemens Healthineers, Germany) | 0.6 mm slice thickness, 70–150 kV tube voltage, rotation speed 0.25s, temporal resolution of 66 ms per segment, slice thickness 0.4-0.6 mm | CoronaryDoc (Shukun Technology software version 6.21) | CoronaryDoc (Shukun Technology software version 6.21) |
| Huang et al. 2024 | 2 centers: a third generation dual-source computed tomography (CT) scanner Siemens Somatom Force and philips integnuity CT  Other center: a 64-slice helical CT scanner (Ingenuity CT, Philips) | Two centers: collimation width (192 × 0.6 mm), tube voltage (100–120 kV), tube current 150-288 mA, slice thickness (0.6 mm), contrast 40−80 mL of iodinated contrast medium (Ultravist, 370 mg iodine/mL, Bayer) was injected at a flow rate of 4−5 mL/s  Other center: tube voltage 120 kV, tube current ranging 150-200 mA or modulated automatically, a tube rotation time of 280 ms, a temporal resolution of 33 ms, and an acquisition phase ranging from 65% to 80%. Reconstruction parameters incorporated a slice thickness of 0.75 or 0.9 mm and a slice increment of 0.5 mm. Intravenously, 40−80 mL of iodinated contrast medium (Ultravist, 370 mg iodine/mL, Bayer) was injected at a flow rate of 4−5 mL/s, immediately followed by a 20 mL saline chaser. | Manually by 2 radiologists using 3D slicer | Pyradiomics |

Supplementary Table 2: Imaging Details of included studies-

FAI: Fat Attenuation Index, BMI: Body Mass Index, NM: Not Mentioned

| **Author** | **n of initial features** | **Final Included radiologic features** | **Include Clinical Features** | **Model Adjustments** | **Initial Feature Selection Process:** | **Interpretability measurements** | **Clinical validity** |
| --- | --- | --- | --- | --- | --- | --- | --- |
| Zhang et al. (2024) | 285 | 15 RCA, 9 LAD, 7 LCX, 15 PCAT radiomics features | LDL-C, hs-CRP, TG | NM | features excluded: radiomics: features with a Spearman’scorrelation coefficient of > 0.9 clinical: variables with p>0.05 in logestic regression | NM | The DCA demonstrated that for predicting MACE in patients with CAD, the overall model had an excellent overall net benefit within the majority of reasonable threshold probabilities with an excellent callibration |
| Miao et al. (2024) | 1037 for each segment | 190 radiomics features including 56 first order , 11 shape, 21 GLCM, 32 GLRLM, 37 GLSZM, 27 GLDM, 6 NGTDM | Age, diastolic pressure, HDL-C, LDLC, TC, TG, HDL, Diastolic pressure, HTN, family history of diabetes | NM | Selection based on significance level (p<0.001) between two groups | Feature importance analysis revealed first order and shape radiomics features as the most important. Among clinical features HTN and age and LDL had the most importance | NM |
| He et al. (2024) | 23 total initial features | PCAT-FAI for RCA, LAD, PCAT-FV RCA  Also CAC score | Age, HTN, smoking status | NM | Feature selected by LASSO regularization | Feature importance by LASSO | According to DCA ensemble had the highest net benefit both in training and validation groups. |
| Chan et al. (2024) | NM | The highest FAI Score (most inflamed artery)  AI risk  Also Plaque burden and severity assessed using the CAD-RADS 2.0 classification, which classifies CAD severity | Diabetes, smoking, hyperlipidemia, and HTN  . | Previous coronary interventions and medications, Age and sex   diabetes, smoking, hyperlipidemia, HTN., Extent of CAD assessed using the CAD-RADS 2.0 classification system | NM | NM | NM |
| Militello et al. (2023) | 93 | First Order(FO), GLCM, GLRLM  GLSZM, GLDM, NGTDM | Tree based feature selected: age, current HTN, statin treatment, vasculopathy.; L1-based and mutual information: age | NM | near zero variance analysis, redundant feature analysis in Mann-Whitney and Fisher exact’s test | Feature importance by: accumulation of the impurity decrease (MDI): Age, Total energy, GLV, GLNN most discriminative. Other clinical features were not discriminative with indicate need for radiomics features. | NM |
| You et al. (2023) | 92 from each region of interest, total 184 | First 30 features by MCMR. Then used GBDT: 12 PCAT radiomics features, 2 EAT radiomics features | Cholesterol, LDL, TG | NM | Maximum relevance minimum redundancy | GBDT for feature importance: | when the threshold probability is >0.43, using the MPCAT-clinical to predict MACE adds more benefit than using the MEAT-clinical in the training cohort, and when the threshold probability is > 0.59, using the MPCAT-clinical to predict MACE adds more benefit than using the MEAT-clinical in the validation cohort |
| Wei He et al. (2024) | NM | FAI  Plaque charactristics: (plaque length, total plaque volume and burden, maximum area stenosis, lipid plaque volume, maximum diameter stenosis, calcified plaque burden and volume, fibrous plaque volume and burden, minimum luminal area, remodeling index | No |  | Significant variables in both univariate and multivariate analysis | Feature importance analysis: FAI, minimal luminal area, total plaque volume, total plaque burden, and lipid plaque burden as the most important features. | NM |
| Huang et Al. (2023) | NM | FAI | No | NA | NA | Feature importance using ablation experiment, | NM |
| Oikonomouet al. (2024) | 1686 (2*843) | 335 features including FAI, Fat radiomics profile | No | Age, sex, SBP, total cholesterol, HDL, DM, smoking, BMI, obstructive disease, scanner type, HRP features presence, calcium score | Only features with pairwise correlation<0.9 using find correlation, caret package, R remained | Feature importance using univariate analysis: (kurtosis, skewness LLL, skewness for RCA as 3 top contributors), overlap between the radiotranscriptomic signatures for adipose tissue inflammation, fibrosis and vascularity and the features included in the FRP also investigated, | NM |
| Zhan, 2024 | 282 (3*94) | 11 including first order, texture, and morpholical features, 4 sselected by LASSO  FAI | Age | NA | P<0.05 on Mann-whitney U test and Spearman’s rank correlation coefficient> 0.9, then by LASSO | NM | Decision curve analysis the nbenefit of predicting MACE in angina pectoris was greater with the radiomics model than with the other models . Calibration curves for the three predictive models showed good agreement between predicted and observed probabilities of adverse events in angina pectoris |
| Huang, 2024 | 851 | 12 features including first order, morpholical, and textural features (GLCM, GLSZM) as Rad-score  Also diameter stenosis, high risk plaque | Age, smoking, HTN, dyslipidemia, DM, use of aspirin, use of ACEi,ARB drugs | NA | Fisher score and pearson ICC<0.85, a feature stability method built using combination of fish score and person_score | Interpretablity by SHAP: the shape-based feature, LeastAxisLength, emerged asmthe most influential predictor. It was followed by the texture feature, Glszm_GrayLevelNonUniformity, serving as a significant predictor of MACE. Glszm_ZoneEntropy was found to be important for the outcome as well Meanwhile, Glszm_SmallAreaLowGreyLevelEmphasis contributed the least among the 12 features | Decision curve analysis: demonstrated a superior overall net benefit, substantiating its enhanced clinical utility for MACE prediction over alternative models in comparison. Normogram had the most net benefit foolowed by CCTA radiologic model. Although radiomics model had better net benefit in lower thrsholds compared to CCTA model. |

Supplementary Table 3: Summary of model features, feature selection method and clinical validity of the models

BMI: Body Mass Index, CAD: Coronary Artery Disease, CAC: Coronary Artery Calcium, DCA: Decision Curve Analysis, EAT: Epicardial Adipose Tissue, FAI: Fat Attenuation Index, GLCM: Gray Level Co-occurrence Matrix, GLSZM: Gray Level Size Zone Matrix, NGTDM: Neighborhood Gray Tone Difference Matrix, HDL: High-Density Lipoprotein, HTN: Hypertension, ICC: Intraclass Correlation Coefficient, LASSO: Least Absolute Shrinkage and Selection Operator, LAD: Left Anterior Descending artery, LCX: Left Circumflex artery, RCA: Right Coronary Artery, MACE: Major Adverse Cardiac Events, SBP: Systolic Blood Pressure, SHAP: SHapley Additive exPlanations, TG: Triglycerides, PCAT: Pericoronary Adipose Tissue, LDL: Low-Density Lipoprotein

|  | |  | | | | Training dataset | | | | | | Test Dataset/ Validation | | | | | |  |
| --- | --- | --- | --- | --- | --- | --- | --- | --- | --- | --- | --- | --- | --- | --- | --- | --- | --- | --- |
| Author | **Feature importance & regularization** | **Model algorithm** | **Feature type** | **Feature specifics** | **Model name** | **AUC** | **Lower 95%CI** | **Higher 95%CI** | **sensitivity** | **specificity** | **accuracy** | **AUC** | **Lower 95%CI** | **Higher 95%CI** | **sensitivity** | **specificity** | **accuracy** | **Validation method** |
| Zhang et al. (2024) | LASSO | ML | Radiomics | PCAT on RCA | RCA-modelx | 0.706 | 0.638 | 0.773 | 0.584 | 0.741 | 0.662 | 0.675 | 0.531 | 0.82 | 0.571 | 0.793 | 0.684 | 10 fold cross validation |
|  | LASSO | ML | Radiomics | PCAT on LCX | LCX-Model | 0.651 | 0.58 | 0.723 | 0.664 | 0.607 | 0.636 | 0.623 | 0.474 | 0.773 | 0.643 | 0.586 | 0.614 |  |
|  | LASSO | ML | Radiomics | PCAT on LAD | LAD-model | 0.679 | 0.609 | 0.748 | 0.699 | 0.625 | 0.662 | 0.664 | 0.517 | 0.811 | 0.785 | 0.517 | 0.649 |  |
|  | LASSO | ML | Radiomics | PCAT | PCAT-model | 0.764 | 0.703 | 0.825 | 0.797 | 0.607 | 0.702 | 0.723 | 0.589 | 0.857 | 0.714 | 0.586 | 0.649 |  |
|  | LASSO | LR | Clincial | Clinical | Cli-model | 0.752 | 0.689 | 0.815 | 0.46 | 0.946 | 0.702 | 0.706 | 0.564 | 0.847 | 0.464 | 0.862 | 0.667 |  |
|  | LASSO | ML Ensemble | Clinical & Radiomics | Clinical & Radiomics | overall model | 0.828 | 0.776 | 0.881 | 0.77 | 0.75 | 0.76 | 0.797 | 0.679 | 0.915 | 0.643 | 0.793 | 0.719 |  |
| Miao et al. (2024) | NA | ML SVM | Clinical | Clinical |  | 0.804 | 0.738 | 0.869 | 0.863 | 0.7324 | 0.799 |  |  |  |  |  |  | NM |
|  |  | ML SVM | Clinical & Radiomics | Clinical & Vessel radiomics |  | 0.941 | 0.902 | 0.981 | 0.941 | 0.95 | 0.944 |  |  |  |  |  |  |  |
|  |  | ML SVM | Clinical & Radiomics | Clinical & Vessel & PCAT |  | 0.949 | 0.913 | 0.986 | 0.952 | 0.951 | 0.951 |  |  |  |  |  |  |  |
|  |  | LR | Clinical | Clinical |  | 0.832 | 0.771 | 0.894 | 0.939 | 0.726 | 0.847 |  |  |  |  |  |  |  |
|  |  | ML DT | Clinical & Radiomics | vessel radiomics |  | 0.885 | 0.831 | 0.938 | 0.915 | 0.839 | 0.882 |  |  |  |  |  |  |  |
|  |  | ML DT | Clinical & Radiomics | Clinical & Vessel radiomics & PCAT |  | 0.885 | 0.831 | 0.938 | 0.915 | 0.855 | 0.889 |  |  |  |  |  |  |  |
|  |  | ML RF | Clinical | Clinical |  | 0.838 | 0.777 | 0.9 | 0.841 | 0.857 | 0.847 |  |  |  |  |  |  |  |
|  |  | ML RF | Clinical & Radiomics | Clinical & Vessel radiomics |  | 0.919 | 0.873 | 0.965 | 0.918 | 0.932 | 0.984 |  |  |  |  |  |  |  |
|  |  | ML RF | Clinical & Radiomics | Clinical & Vessel & PCAT |  | 0.917 | 0.87 | 0.964 | 0.908 | 0.947 | 0.924 |  |  |  |  |  |  |  |
| He et al. (2024) | LASSO | LR | Clinical & Radiomics | Clinical & Radiomics |  | 0.89 | 0.85 | 0.93 | 0.82 | 0.8 | 0.81 | 0.84 | 0.79 | 0.89 | 0.73 | 0.76 | 0.75 | 5 fold cross validation |
|  | LASSO | FS | Clinical & Radiomics | Clinical & Radiomics |  | 0.62 | 0.54 | 0.7 | 0.67 | 0.5 | 0.55 | 0.61 | 0.53 | 0.69 | 0.67 | 0.5 | 0.55 |  |
|  | LASSO | ML AdaBoost | Clinical & Radiomics | Clinical & Radiomics |  | 0.95 | 0.92 | 0.98 | 0.67 | 0.96 | 0.87 | 0.82 | 0.76 | 0.88 | 0.48 | 0.91 | 0.78 |  |
|  | LASSO | ML GNB | Clinical & Radiomics | Clinical & Radiomics |  | 0.88 | 0.83 | 0.93 | 0.63 | 0.91 | 0.83 | 0.86 | 0.8 | 0.92 | 0.58 | 0.93 | 0.82 |  |
|  | LASSO | ML LDA | Clinical & Radiomics | Clinical & Radiomics |  | 0.9 | 0.85 | 0.95 | 0.63 | 0.93 | 0.84 | 0.85 | 0.79 | 0.91 | 0.57 | 0.91 | 0.81 |  |
|  | LASSO | ML SVM | Clinical & Radiomics | Clinical & Radiomics |  | 0.93 | 0.89 | 0.97 | 0.78 | 0.95 | 0.9 | 0.87 | 0.8 | 0.94 | 0.57 | 0.89 | 0.79 |  |
|  | LASSO | ML Ensemble | Clinical & Radiomics | Clinical & Radiomics |  | 0.94 | 0.91 | 0.97 | 0.87 | 0.88 | 0.87 | 0.93 | 0.9 | 0.96 | 0.85 | 0.89 | 0.88 |  |
| Militello et al. (2023) | L1 | ML RF | Clinical | Clinical |  | 0.684 | 0.596 | 0.772 | 0.642 | 0.621 | 0.628 |  |  |  |  |  |  | 5 fold cross validation |
|  | L1 | ML RF | Radiomics | Radiomics |  | 0.741 | 0.66 | 0.822 | 0.691 | 0.635 | 0.659 |  |  |  |  |  |  |  |
|  | L1 | ML RF | Clinical & Radiomics | Clinical & Radiomics |  | 0.793 | 0.716 | 0.87 | 0.74 | 0.708 | 0.719 |  |  |  |  |  |  |  |
|  | Tree | ML RF | Clinical | Clinical |  | 0.684 | 0.596 | 0.772 | 0.642 | 0.621 | 0.628 |  |  |  |  |  |  |  |
|  | Tree | ML RF | Radiomics | Radiomics |  | 0.819 | 0.745 | 0.893 | 0.767 | 0.681 | 0.72 |  |  |  |  |  |  |  |
|  | Tree | ML RF | Clinical & Radiomics | Clinical & Radiomics |  | 0.819 | 0.749 | 0.889 | 0.766 | 0.713 | 0.735 |  |  |  |  |  |  |  |
|  | Mutual information | ML RF | Clinical | Clinical |  | 0.666 | 0.585 | 0.747 | 0.646 | 0.613 | 0.626 |  |  |  |  |  |  |  |
|  | Mutual information | ML RF | Radiomics | Radiomics |  | 0.803 | 0.723 | 0.879 | 0.762 | 0.672 | 0.713 |  |  |  |  |  |  |  |
|  | Mutual information | ML RF | Clinical & Radiomics | Clinical & Radiomics |  | 0.82 | 0.744 | 0.896 | 0.77 | 0.716 | 0.739 |  |  |  |  |  |  |  |
| You et al. (2023) | GBDT | ML LR | Radiomics | PCAT | MPCAT | 0.69 | 0.616 | 0.763 | 0.604 | 0.68 | 0.69 | 0.703 | 0.591 | 0.816 | 0.604 | 0.682 | 0.644 | External validation cohort |
|  | GBDT | ML LR | Radiomics | Epicardial adipose | MEAT | 0.543 | 0.463 | 0.623 | 0.446 | 0.56 | 0.543 | 0.538 | 0.414 | 0.661 | 0.446 | 0.568 | 0.482 |  |
|  | GBDT | LR | Clinical | Clinical | Mclinical | 0.75 | 0.682 | 0.818 | 0.713 | 0.68 | 0.75 | 0.748 | 0.645 | 0.852 | 0.713 | 0.636 | 0.644 |  |
|  | GBDT | ML LR | Clinical & Radiomics | Clinical & Radiomics | MPCAT-clinical | 0.782 | 0.719 | 0.846 | 0.683 | 0.74 | 0.782 | 0.781 | 0.684 | 0.878 | 0.683 | 0.682 | 0.69 |  |
|  | GBDT | ML LR | Clinical & Radiomics | Clinical & Radiomics | MEAT-clinical | 0.747 | 0.678 | 0.815 | 0.713 | 0.68 | 0.747 | 0.745 | 0.641 | 0.849 | 0.713 | 0.636 | 0.643 |  |
| He et al. (2024) | NM | LR | Radiomics | Plaque charactristics |  | 0.76 | 0.70 | 0.82 |  |  |  |  |  |  |  |  |  |  |
|  | NM | LR | Radiomics | FAI |  | 0.93 | 0.89 | 0.96 |  |  |  |  |  |  |  |  |  |  |
|  | NM | ML XGBoost | Radiomics | Plaque characteristics |  | 0.92 | 0.88 | 0.96 | 0.86 | 0.85 | 0.85 | 0.84 | 0.68 | 0.99 | 0.83 | 0.82 | 0.73 | 10 fold cross validation |
|  | NM | ML XGBoost | Radiomics | FAI + Plaque |  | 0.99 | 0.98 | 1 | 0.97 | 0.93 | 0.95 | 0.94 | 0.88 | 1 | 0.92 | 0.89 | 0.85 |  |
| Oikonomou et al. (2019) | RFE | ML RF | Radiomics | PCAT |  |  |  |  |  |  |  | 0.774 | 0.622 | 0.926 |  |  |  | Only testset |
| Huang et al. (2023) | NM | ML LR | Radiomics | PCAT + Plaque |  |  |  |  |  |  |  | 0.744 | 0.658 | 0.83 | 0.48 | 0.893 | 0.687 | NM |
|  | DL FC CNN | | Radiomics | PCAT + Plaque of Proximal-stream |  |  |  |  |  |  |  | 0.658 | 0.565 | 0.751 | 0.88 | 0.533 | 0.707 |  |
|  | DL FC CNN | | Radiomics | PCAT + Plaque of lesion stream |  |  |  |  |  |  |  | 0.875 | 0.81 | 0.94 | 0.713 | 0.853 | 0.833 |  |
|  | DL FC CNN single stream | | Radiomics | PCAT + Plaque |  |  |  |  |  |  |  | 0.748 | 0.663 | 0.833 | 0.773 | 0.8 | 0.787 |  |
|  | DL FC CNN two stream | | Radiomics | PCAT + Plaque |  |  |  |  |  |  |  | 0.922 | 0.869 | 0.975 | 0.893 | 0.827 | 0.86 |  |
|  | DL Two-stream CNN + CFF + single linear FC | | Radiomics | PCAT + Plaque | Two-stream |  |  |  |  |  |  | 0.919 | 0.866 | 0.972 | 0.867 | 0.92 | 0.893 |  |
|  | DL CNN | | Radiomics | PCAT + Plaque | TSCFE-CFF |  |  |  |  |  |  | 0.969 | 0.938 | 1 | 0.907 | 0.92 | 0.913 |  |
| Weisheng Zhan (2024) | LASSO | ML RF | Radiomics | PCAT for LAD, LCX, RCA |  | 0.83 | 0.75 | 0.913 | 0.63 | 0.86 | 0.81 | 0.71 | 0.54 | 0.87 | 0.42 | 0.87 | 0.79 | Internal validation |
|  | LASSO | ML LR | Clinical | Clinical |  | 0.81 | 0.74 | 0.88 | 0.74 | 0.7 | 0.71 | 0.67 | 0.53 | 0.81 | 0.67 | 0.62 | 0.63 |  |
|  | LASSO | ML RF | Radiomics | FAI |  | 0.71 | 0.61 | 0.81 | 0.51 | 0.77 | 0.71 | 0.54 | 0.35 | 0.73 | 0.25 | 0.83 | 0.74 |  |
| Chan et al. (2024) | NM | Ensemble | Clinical & Radiomics | FAI Score, CAD-RADS 2.0, and QRISK3 | Overall model | 0.854 | 0.851 | 0.857 |  |  |  |  |  |  |  |  |  | NM |
|  |  | LR | Clinical |  | QRISK3 | 0.831 | 0.83 | 0.832 |  |  |  |  |  |  |  |  |  |  |
|  |  | ML Ensemble | Clinical & Radiomics | Non-Obstructive CAD Group |  | | | | | | | | 0.816 | 0.812 | 0.82 |  |  |  |
|  |  | ML Ensemble | Clinical & Radiomics | Obstructive CAD Group |  | | | | | | | | 0.773 | 0.759 | 0.787 |  |  |  |
| Huang et al. (2024) | NM | ML Ensemble | Overall | Normogram |  |  |  |  |  |  |  | 0.966 |  |  | 0.85 | 0.98 | 0.944 | Internal+ external validation |
|  |  | LR | Clinical |  |  |  |  |  |  |  |  | 0.84 |  |  | 0.65 | 0.922 | 0.897 |  |
|  |  | ML | Radiologic |  |  |  |  |  |  |  |  | 0.817 |  |  | 0.55 | 0.922 | 0.825 |  |
|  |  | ML | Radiomics |  |  |  |  |  |  |  |  | 0.901 |  |  | 0.75 | 0.961 | 0.833 |  |

Supplementry Table 4: summary of predictive models used in the included studies;

AUC: Area Under the Curve, CAD: Coronary Artery Disease, FAI: Fat Attenuation Index, CNN: Convolutional Neural Network, DL: Deep Learning, GBDT: Gradient Boosted Decision Trees, GNB: Gaussian Naive Bayes, LAD: Left Anterior Descending artery, LASSO: Least Absolute Shrinkage and Selection Operator, LCX: Left Circumflex artery, LR: Logistic Regression, ML: Machine Learning, MPCAT: Machine Learning Pericoronary Adipose Tissue, PCAT: Pericoronary Adipose Tissue, RF: Random Forest, RFE: Recursive Feature Elimination, SVM: Support Vector Machine, TSCFE-CFF: Two-Stream Convolutional Feature Extractor Combined with Compact Feature Fusion

| **study** | **Participants** | | **Predictors** | | **Outcome** | | **Analysis** | **Overall** |
| --- | --- | --- | --- | --- | --- | --- | --- | --- |
|  | **Risk of bias** | **Applicability** | **Risk of bias** | **Applicability** | **Risk of bias** | **Applicability** |  |  |
| Oikonomou, 2019 | Low | Low Concern | Low | Low Concern | Low | Low Concern | Low | Low |
| Miao, 2024 | Some concerns | High Concern | Low | Low Concern | High | Some Concerns | High | High |
| Wei He, 2024 | Some concerns | Low Concern | Low | Low Concern | High | Low Concern | High | High |
| You, 2023 | Low | Low Concern | Low | Low Concern | Low | Low Concern | Low | Low |
| Zhan, 2024 | Some concerns | Low Concern | Low | Low Concern | Low | Low Concern | High | High |
| Zhang, 2024 | Low | Low Concern | Low | Low Concern | Low | Low Concern | Low | Low |
| Chan, 2024 | Low | Low Concern | Low | Low Concern | Low | Low Concern | Low | Low |
| He, 2024 | Some concerns | High Concern | Low | Low Concern | Low | Low Concern | High | High |
| Militello, 2023 | Some concerns | High Concern | Low | Low Concern | High | Low Concern | Some concerns | High |
| Huang, 2023 | Some concerns | Low Concern | Low | Low Concern | Low | Low Concern | High | High |
| Huang, 2024 | Low | Low Concern | Low | Low Concern | Low | Low Concern | Low | Low |

Supplementary Table 5: risk of bias assessment and applicability of included articles regarding 5 different domains according to PROBAST-AI

**Supplementary text for result:**
In the analysis of the best models, publication bias was assessed using several statistical methods. The classic fail-safe N indicated that 345,592 null studies would be required to nullify the observed effect (z = 307.95, p < 0.0001), corresponding to 24,685.1 missing studies for each observed study. Orwin’s fail-safe N showed that the mean effect size among missing studies needed to be 0 to shift the combined effect estimate below the specified threshold. Begg and Mazumdar’s rank correlation test showed no significant publication bias (Kendall’s tau b = -0.2967, p = 0.0697 for a 1-tailed test; p = 0.1394 for a 2-tailed test). Egger’s test of the intercept revealed no significant publication bias (B0 = 2.5494, 95% CI = -2.6065 to 7.7053; t = 1.0773, p = 0.1513 for a 1-tailed test; p = 0.3025 for a 2-tailed test). Duval and Tweedie’s Trim and Fill analysis, using a fixed effects model, suggested that two studies were missing, yielding an adjusted point estimate of 0.8559 (95% CI = 0.8532 to 0.8586) compared to the observed estimate of 0.8674 (95% CI = 0.8646 to 0.8703). Under the random effects model, the adjusted estimate was 0.8576 (95% CI = 0.8091 to 0.9062), compared to the observed estimate of 0.8789 (95% CI = 0.8316 to 0.9263). Overall, no significant evidence of publication bias was found, although the trim and fill analysis suggested a small adjustment to the combined effect size. Meanwhile, in the analysis of all models, Egger’s test indicated no significant publication bias for the combined effect size (intercept B₀ = -0.02847; 95% CI, -1.29016 to 1.23322; t = 0.04496; p = 0.48213 [1-tailed], p = 0.96426 [2-tailed]). Notably, Duval and Tweedie’s trim-and-fill method identified no missing studies under either the fixed-effects model (combined estimate = 0.83384; 95% CI, 0.83293 to 0.83475) or the random-effects model (combined estimate = 0.82169; 95% CI, 0.81022 to 0.83316), as these estimates remained unchanged. The classic fail-safe N analysis revealed a fail-safe N of 5,579,546, indicating that 5,579,546 missing 'null' studies would be required to render the observed effect size non-significant (p > 0.05). This equates to 73,415.1 missing studies per observed study to nullify the effect. Orwin's fail-safe N could not be computed due to incompatible criterion settings for effect size.
